# Reversible [4 + 2] cycloaddition reaction of 1,3,2,5-diazadiborinine with ethylene†
†Electronic supplementary information (ESI) available: Experimental and calculation details, and crystallographic information for **2**, **4a**, **4b**, **4d**, and **6**. CCDC 1418724–1418728. For ESI and crystallographic data in CIF or other electronic format see DOI: 10.1039/c5sc03174e


**DOI:** 10.1039/c5sc03174e

**Published:** 2015-09-15

**Authors:** Di Wu, Rakesh Ganguly, Yongxin Li, Sin Ni Hoo, Hajime Hirao, Rei Kinjo

**Affiliations:** a Division of Chemistry and Biological Chemistry , School of Physical and Mathematical Sciences , Nanyang Technological University , Singapore 637371 , Singapore . Email: rkinjo@ntu.edu.sg ; Email: hirao@ntu.edu.sg; b NTU-CBC Crystallography Facility , Nanyang Technological University , Singapore 637371 , Singapore

## Abstract

Under ambient conditions, a [4 + 2] cycloaddition reaction of 1,3,2,5-diazadiborinine **1** with ethylene afforded a bicyclo[2.2.2] derivative **2**, which was structurally characterized. The cyclization process was found to be reversible, and thus retro-[4 + 2] cycloaddition reproduced **1** quantitatively, concomitant with the release of ethylene. Compound **1** reacted regio-selectively and stereo-selectively with styrene derivatives and norbornene, respectively, and these processes were found to be reversible too. Computational studies determined the reaction pathways which were consistent with the regio-selectivity observed in the reaction of styrene, and the reaction was suggested to be essentially concerted but highly asynchronous.

## Abstract 2

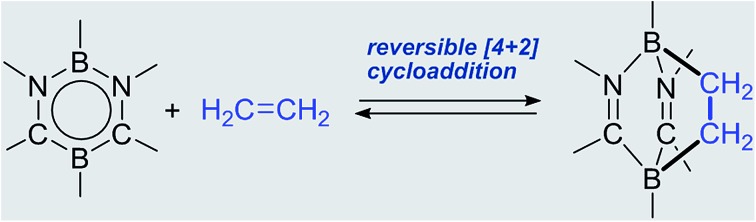
Under ambient conditions, a [4 + 2] cycloaddition reaction of 1,3,2,5-diazadiborinine **1** with ethylene afforded a bicyclo[2.2.2] derivative **2**, which was structurally characterized.

## Introduction

Reversible coordination processes between a metal center and olefins are of paramount significance in transition metal catalysis.^1^ The labile yet attractive metal-olefin interactions can be accounted for by the Dewar–Chatt–Duncanson model;^2^ this model rationalizes, for example, the bonding mode in Zeise's salt [Cl_3_Pt(η^2^-C_2_H_4_)]K,^3^ in which the donation of π-electrons from ethylene to an unoccupied d-orbital of the metal and π-backdonation of d-electrons from the metal to the π*-orbital of ethylene occur concurrently.^4^ Such simultaneous donor–acceptor interactions give unique chemical properties to transition metal complexes.

**Fig. 1:**
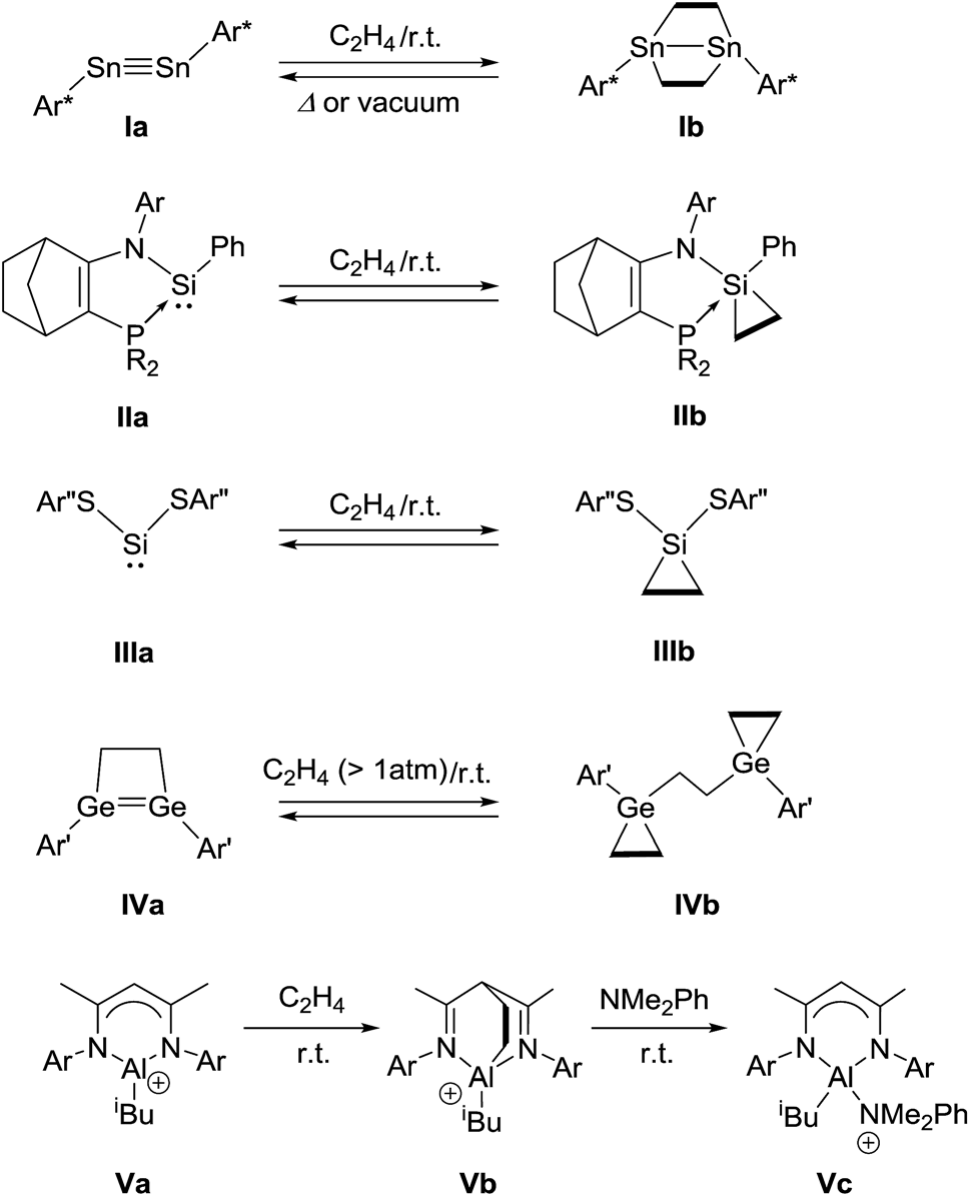
Examples of reversible cycloaddition reactions between main group compounds and ethylene.

In recent years, it has been amply demonstrated that main group elements can behave as transition metals,^5^*e.g.*, in unique activation reactions of ethylene^6^ as well as other olefins^7^ where several systems were found to bind to olefins reversibly (Fig. 1). In 2009, Power *et al.* reported a cycloaddition reaction of distannyne **Ia** with two equivalents of ethylene, which afforded a bicycle[2.2.0] derivative **Ib**.8*a* Remarkably, **Ib** readily released ethylene molecules *via* thermal retro-cycloaddition under reduced pressure or upon standing at 25 °C, to reproduce **Ia**. Kato, Baceiredo, and co-workers demonstrated that ethylene could be incorporated onto phosphonium sila-ylide, giving rise to silirane **IIb**.^9^ The formation of **IIb** depended on the ethylene pressure, and indeed, **IIb** reverted to **IIa** after a decrease in ethylene pressure. Recently, Tuononen and Power *et al.* reported the reversible binding of ethylene to two-coordinate acyclic silylene **IIIa** under ambient conditions, which generated sililane **IIIb** with a tetra-coordinate silicon atom.^10^ Very recently, Tokitoh and Sasamori *et al.* reported that 1,2-digermacyclobutene **IVa** underwent sequestration of two ethylene molecules to produce **IVb**.^11^ Retro-conversion of **IVb** to **IVa** was observed by reducing the ethylene pressure. In these reactions, the formation of the cycloadducts is inferred to involve a [1 + 2] interaction between the heavier group 14 element center and the C=C bond of ethylene in the initial step. With a group 13 compound, Jordan *et al.* reported that the cationic aluminum β-diketiminate derivative **Va** and ethylene underwent a [4 + 2] cycloaddition reaction, yielding the cycloadduct **Vb**, which was characterized only by NMR spectroscopy.^12^ Although **Vb** did not release ethylene even under vacuum, the addition of *N*,*N*-dimethylaniline to a solution of **Vb** allowed ethylene elimination concomitant with the formation of **Vc** at room temperature. Additive-free reversible [4 + 2] cyclization between a 6π-compound and ethylene still remains highly challenging.^13^

Recently, we reported the preparation of aromatic 1,3,2,5-diazadiborinine **1**,14*a* which formally involves a neutral tricoordinate boron center surrounded by eight electrons.5a,14b–h Preliminary studies indicated the unique reactivity of **1** toward unsaturated compounds such as carbon dioxide, forming bicyclo[2.2.2] derivatives, which prompted us to investigate the reactions of **1** with ethylene and other non-activated olefins in detail. Herein, we report the reversible cycloaddition reactions of **1** with various alkenes. We also describe the single-crystal X-ray diffraction analysis of the cycloadducts, kinetic experiments, and computational studies.

## Results and discussion, experimental

### Reaction of **1** with ethylene

**Scheme 1:**
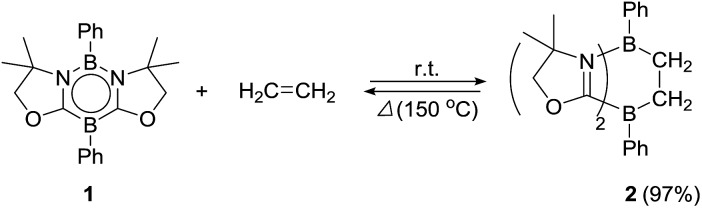
Reversible [4 + 2] cycloaddition reaction between **1** and ethylene.

**Fig. 2:**
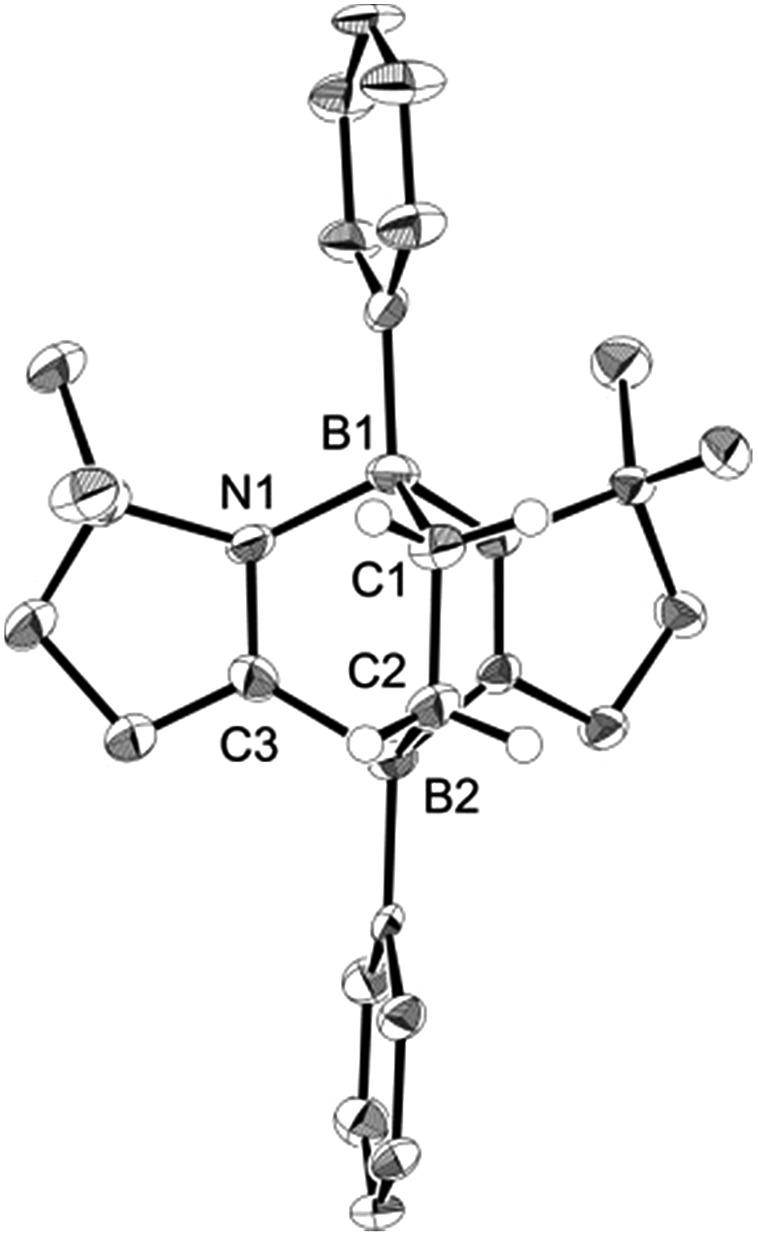
Solid-state structure of **2**. Thermal ellipsoids were set at the 50% probability level. Hydrogen atoms except for those on the C1 and C2 atoms are omitted for clarity. Selected bond lengths [Å] and angles [°]: B1–C1 1.653(5), B1–N1 1.588(5), B2–C2 1.671(5), B2–C3 1.593(5), C1–C2 1.550(4), C3–N1 1.302(4); N1–B1–C1 104.0(3), B1–C1–C2 110.4(3), C1–C2–B2 113.3(3), C2–B2–C3 100.7(3).

Ethylene was introduced into a benzene solution of **1** at 1 bar and the reaction mixture was stirred at ambient temperature for 3 h. After the removal of benzene and ethylene *in vacuo*, compound **2** was obtained as a white power in 97% yield (Scheme 1). In the ^11^B NMR spectrum of **2**, two peaks were detected at –16.0 ppm and 1.6 ppm that are shifted upfield with respect to those (7.3 ppm and 24.9 ppm) for the corresponding boron atoms in **1**.^14^ Recrystallization from a saturated benzene solution at room temperature afforded single crystals of **2**, and the solid-state structure was confirmed by X-ray diffraction analysis (Fig. 2). Both of the boron centers are tetra-coordinated, indicating sp^3^ hybridization. The B1–C1 (1.653(5) Å) and B2–C2 (1.671(5) Å) distances are significantly longer than the typical B–C single bond (1.59 Å). The long C1–C2 (1.550(4) Å) distance is an indication of a C(sp^3^)–C(sp^3^) single bond. There is a lengthening of the B1–N1 (1.588(5) Å) and B2–C3 (1.593(5) Å) bonds and a shortening of the C3–N1 (1.302(4) Å) bond, compared to those of **1**.^14^ Compound **2** is stable at room temperature, both in the solid state and in solution. However, upon heating a C_6_D_6_ solution of **2** at 150 °C, a clean ring opening occurred, giving rise to **1** quantitatively. Note that the Diels–Alder reaction between benzene and ethylene as well as the retro Diels–Alder reaction have scarcely been described thus far,^15^ despite the extensive application of the elementary reactions in organic synthesis.^16^

### Reaction of **1** with styrene derivatives

**Scheme 2:**
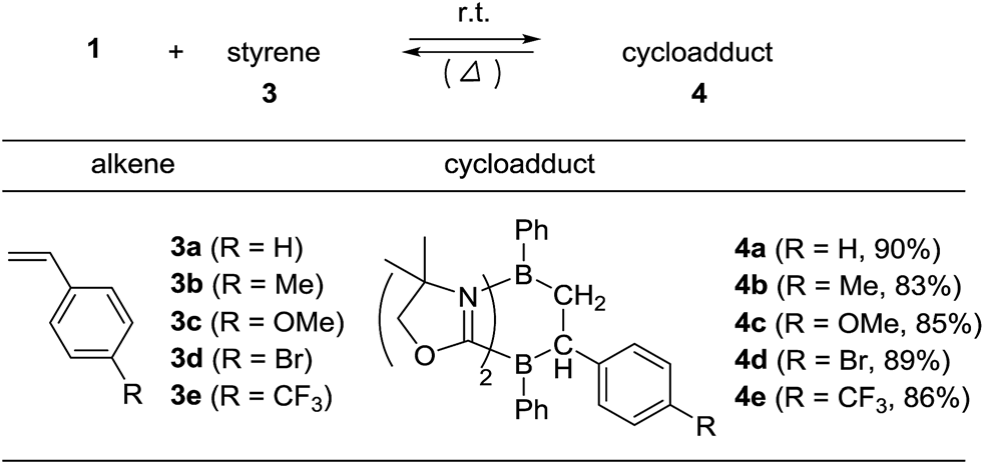
Regio-selective [4 + 2] cycloaddition reactions between **1** and styrenes **3a–e**.

**Fig. 3:**
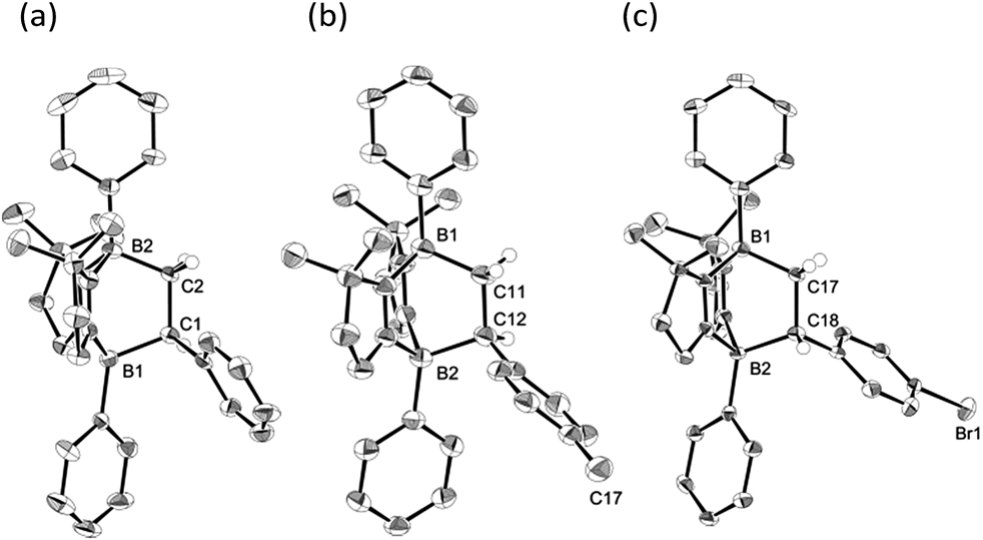
Solid-state structures of **4a** (a), **4b** (b) and **4d** (c). Thermal ellipsoids were set at the 50% probability level. Most of the hydrogen atoms are omitted for clarity.

To examine the scope of the cycloaddition reaction, we employed styrene derivatives **3a–e** as alkene substrates (Scheme 2). A slight excess (2.0 eq.) of styrene **3a** was added to a C_6_D_6_ solution of **1** in a J. Young NMR tube, and the reaction was monitored by NMR spectroscopy. This reaction was drastically slower than that of ethylene, with full conversion of **1** observed only after 12 h at room temperature. After the workup, **4a** was isolated as a white powder in 90% yield. In the ^11^B NMR spectrum of **4a**, two singlets appeared at –14.3 ppm and 0.8 ppm, which are comparable to those (–16.0 ppm and 1.6 ppm) of **2**. X-ray diffraction analysis revealed the bicyclo[2.2.2] structure of **4a** and confirmed that the *C*H_2_ and the *C*HPh carbons of **3a** selectively bind to the boron between two nitrogen atoms and the boron between two carbon atoms in **1**, respectively (Fig. 3a). Remarkably, retro [4 + 2] cycloaddition of **4a** was observed at ambient conditions. Thus, when isolated **4a** was re-dissolved in C_6_D_6_ and the solution was kept at ambient temperature, partial regeneration of **1** and **3a** was detected in the ^1^H NMR spectrum. Meanwhile, the full conversion of **4a** into **1** and **3a** was confirmed upon heating the solution at 110 °C. Cyclization reactions of **1** with *para*-substituted styrene derivatives **3b–e** also proceeded cleanly at room temperature, and the corresponding [4 + 2] cycloadducts **4b–e** were obtained. Analogous to **4a**, the bicyclo[2.2.2] structures of **4b** and **4d** formed *via* regio-selective [4 + 2] cycloaddition were confirmed by X-ray diffraction studies (Fig. 3b and c). We also confirmed that **4b–e** underwent retro [4 + 2] cycloaddition reactions at 110–150 °C, to afford **1** and **3b–e** in 80–100% yields (see the ESI†).

**Fig. 4:**
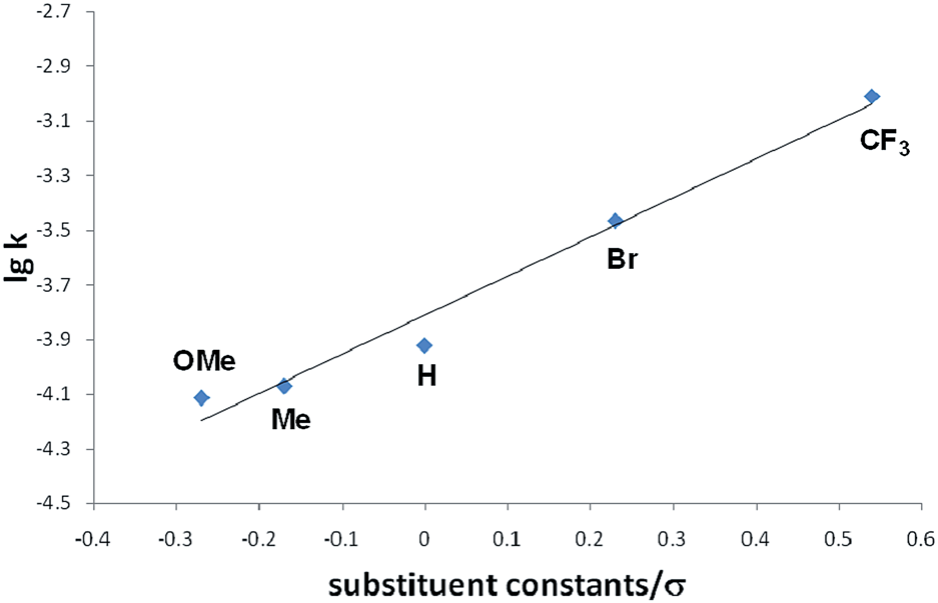
Hammett plot for the reactions of **1** with *para*-substituted styrenes **3a–e** in THF-d_8_ at room temperature.

Interestingly, we noted that the cyclization reaction proceeded faster with styrenes bearing an electron-withdrawing group under the same reaction conditions. Thus, while the reactions with **3a–c** required more than 12 h for the full conversion into the cycloadducts **4a–c**, the reactions with **3d** and **3e** completed within 6 h and 3 h, respectively. Indeed, the equilibrium constant for the reversible cycloaddition highly depends on the *para*-substituent of the styrene derivative (Table 1). To evaluate the substituent effect on the cycloaddition reaction, we checked the Hammett correlation for the reaction of **1** with **3a–e**.^17^ The rate constants *k* were measured based on the conversion rate of **1** using its integral values in the ^1^H NMR spectra (see the ESI†). Fig. 4 shows a linear correlation between log *k* and the substituent constants with *ρ* of +1.43, which is in line with the sensitivity of the cycloaddition reaction to the inductive effect of the substituents.

**Table 1:** Equilibrium constants (*K*_4–1_) at room temperature

Styrene	**3a**	**3b**	**3c**	**3d**	**3e**
*K*_4–1_	1.148 × 10^3^	0.601 × 10^3^	0.457 × 10^3^	8.741 × 10^3^	2.011 × 10^4^

### Computational study on reaction pathway

**Fig. 5:**
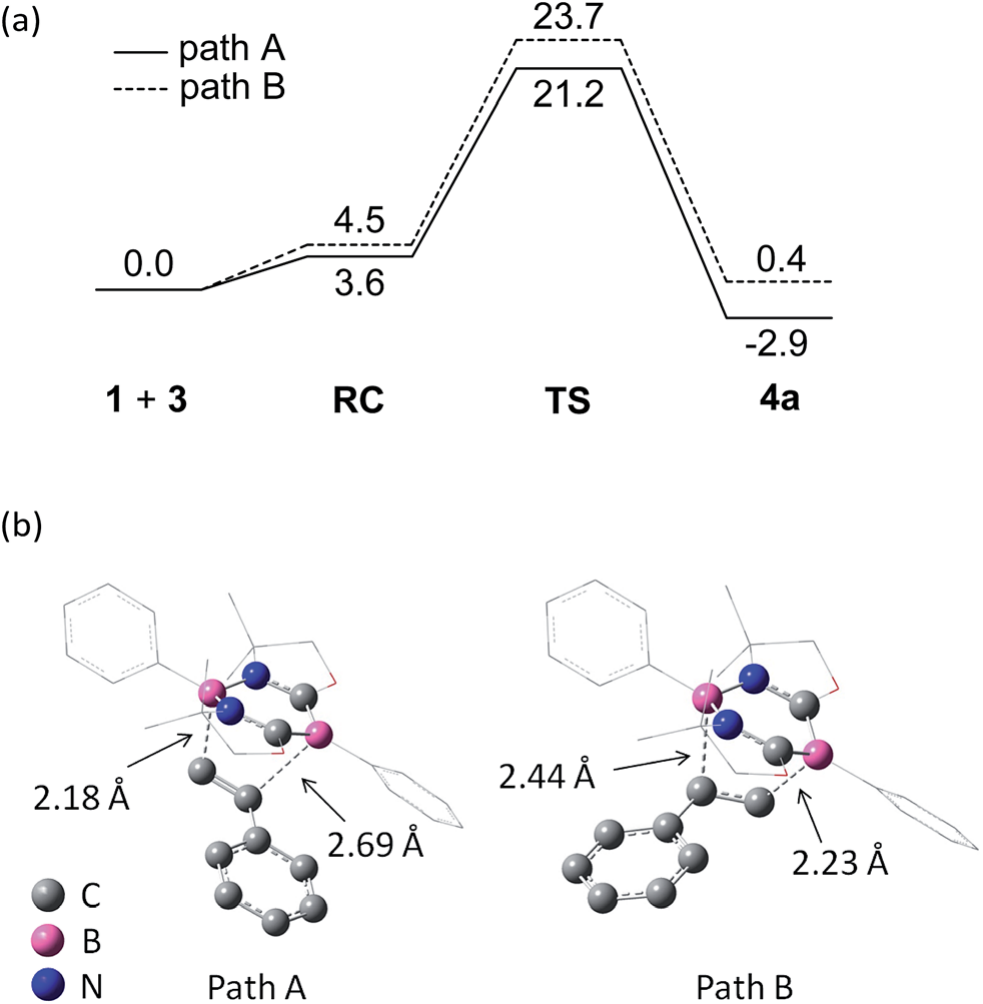
(a) DFT-calculated free energy profiles (kcal mol^–1^) for the proposed mechanism of the [4 + 2] cycloaddition reaction between **1** and ethylene. The optimized structures were obtained at the M06-2X/6-31G* level. The Δ*G* values in C_6_H_6_ at 298 K were obtained from the M06-2X/6-31G* gas-phase harmonic frequencies and the energy in C_6_H_6_ calculated with the def2-TZVP basis set. (b) Transition-state geometries.

To gain insight into the reaction mechanism, pathways for the cycloaddition reaction between **1** and styrene **3a** were explored theoretically using DFT calculations at the M06-2X(SCRF, solvent = benzene)/def2-TZVP//M06-2X/6-31G* level of theory, where the M06-2X functional accounts for the dispersion effects.^18^ We attempted to obtain pathways for the concerted and stepwise mechanisms. However, only concerted pathways could be determined (Fig. 5a), which suggests that the reaction proceeds in a concerted fashion. We could determine two possible pathways for the concerted reaction, namely, a favored pathway (path A) leading to the experimentally observed product and a less favored pathway (path B). Stephan *et al.* demonstrated a stepwise activation of olefin with an intermolecular frustrated Lewis pair system.^19^ Thus, by employing a Lewis acidic borane tethered to a vinyl group through an alkyl chain, they experimentally confirmed that a unique borane–olefin van der Waals (vdW) complex was formed prior to nucleophilic attack by a Lewis base. In our system, a similar vdW interaction could not be observed at the stage of a reactant complex (RC). An important steric reason appears to be that the phenyl ring attached to the Lewis acidic boron atom in the N–B–N moiety is perpendicular to the diazadiborinine ring, and thus the phenyl ring hinders the full coordination of an olefin molecule onto the boron. Moreover, both Lewis acidic and Lewis basic boron centers are present in **1** and interact with each other in the 6π-system, which could allow the cooperative activation of olefin.

**Fig. 6:**
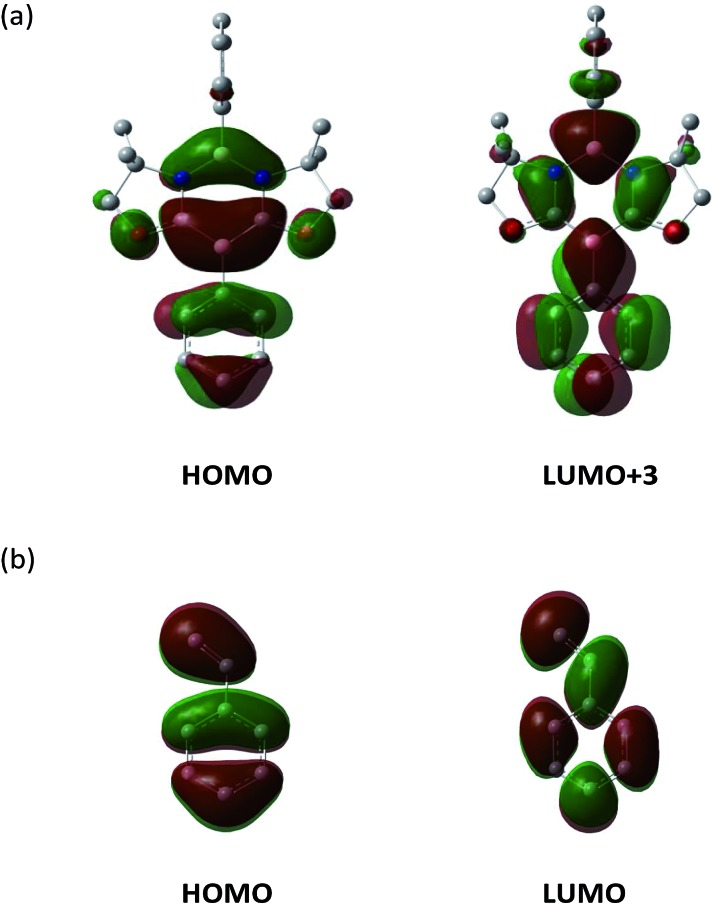
Key frontier orbitals of **1** (a) and styrene **3a** (b).

Consistent with the experimental observation, path A has a lower energy barrier. The computationally estimated activation parameters for the [4 + 2] cycloaddition process on path A (Δ*H*^‡^ = 6.6 kcal mol^–1^, Δ*S*^‡^ = –48.9 e.u., Δ*G*^‡^_(298)_ = 21.2 kcal mol^–1^) agreed reasonably with the experimental values (Δ*H*^‡^ = 11.6 ± 0.9 kcal mol^–1^, Δ*S*^‡^ = –37.6 ± 3.0 e.u., Δ*G*^‡^_(298)_ = 22.8 ± 1.8 kcal mol^–1^). The reaction on path A was calculated as slightly exergonic, consistent with the fact that heating is necessary for the retro-conversion (Scheme 2). It is interesting to note that although the reaction is essentially concerted, the transition state (TS) geometries shown in Fig. 5b suggest that the reaction is highly asynchronous. On path A, one of the two forming B–C bonds has a distance of 2.18 Å, whereas the other B–C bond is longer (2.69 Å). The same trend was also observed recently by Liu *et al.*^20^ The short distance of the former B–C bond reflects an efficient orbital interaction between the LUMO+3 of **1** and the HOMO of styrene **3a** which has a large amplitude on the terminal carbon (Fig. 6). In addition to the orbital interaction, the effects of exchange and electrostatic interactions should play important roles in determining the regioselectivity. The exchange repulsion between the HOMOs of styrene **3a** and **1** may be reduced in this asynchronous configuration, because the HOMO of **1** has a significant amplitude on the C–B–C moiety and the phenyl ring substituted on the boron atom. Furthermore, as shown in Fig. S4-2,† the boron atoms at the N–B–N and C–B–C moieties in **1** have positive (+0.35) and negative (–0.18) charges, respectively, whereas the terminal carbon (–0.39) in styrene **3a** is more electronegative than the internal carbon (–0.12). Therefore, the TS for path B will feel a larger electrostatic repulsion. In terms of orbital interaction, path B should be favorable because it can form an efficient HOMO (**1**)–LUMO (**3a**) interaction (Fig. 6). However, the stabilization arising from this orbital interaction is outweighed by the above-mentioned repulsive effects. It is inferred that in addition to the repulsion at the reaction centers, the steric repulsion between the phenyl ring of **3a** and the two dimethyl moieties (–C*Me*_2_–) of **1** in the TS of path B may also contribute to the distinct regio-selectivity in this reaction (Fig. 5b).

### Reaction of **1** with norbornene

**Scheme 3:**
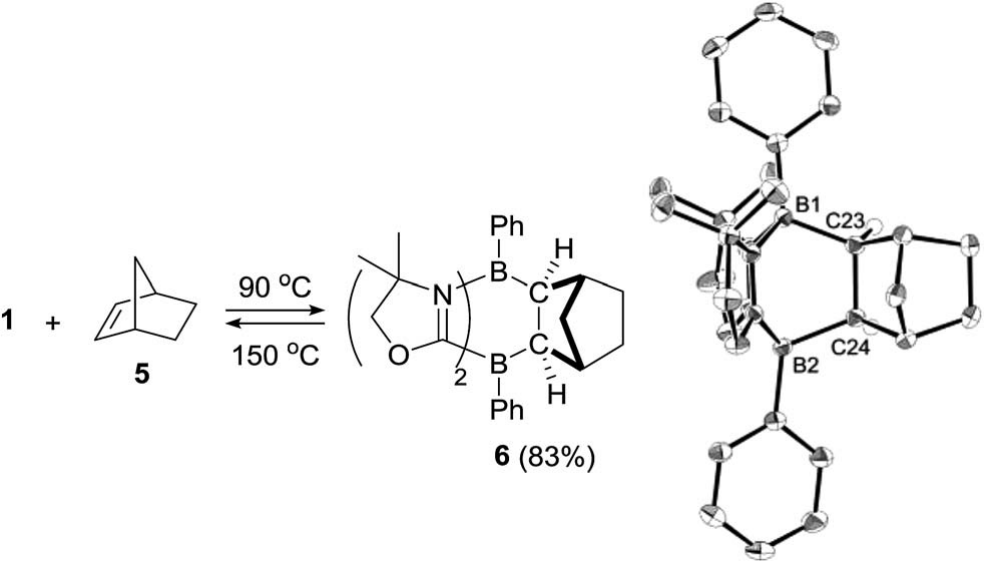
Stereo-selective [4 + 2] cycloaddition reaction between **1** and norbornene **5** and its retro-conversion.

We also investigated the limitations of the substrate scope. Cyclization reactions of **1** with cyclic and acyclic internal alkenes such as 1,1-bis(trimethylsilyl)ethene, *trans*-diphenylethene, cyclohexene, cyclohexadiene did not proceed even under heating conditions (r.t. ∼150 °C), presumably because of steric hindrance. By contrast, the reaction of **1** with norbornene **5**, which features a strained bicyclic skeleton, underwent stereo-selective [4 + 2] cycloaddition at 90 °C, and **6** was obtained as a solo product in 83% yield (Scheme 3). The stereoselectivity of the reaction originated probably from the fact that the steric hindrance of the CH_2_ moiety is smaller than that of the CH_2_CH_2_ part of **5**. Retro-[4 + 2] cycloaddition of **6** proceeded thermally by heating at 150 °C, which cleanly reproduced **1** and **5**.

## Conclusions

In summary, we have demonstrated a [4 + 2] cycloaddition reaction between **1** and ethylene which afforded the bicyclo[2.2.2] compound **2** at room temperature. We also showed regio-selective and stereo-selective [4 + 2] cycloadditions of **1** with styrene derivatives and norbornene, respectively. All of the cycloadducts underwent retro-[4 + 2] cycloaddition reactions that reproduced **1** and the corresponding alkenes quantitatively. Computational studies using DFT calculations showed that the transition state for the cycloaddition is concerted, but highly asynchronous, and provided explanations for the observed regioselectivity. Studies on the reversible cycloaddition of **1** with substrates featuring other unsaturated bonds, and its application^20^ are currently under investigation in our laboratory.

## Supplementary Material

Supplementary information

Crystal structure data
