# Supplementary material for: Reversible [4 + 2] cycloaddition reaction of 1,3,2,5-diazadiborinine with ethylene
Source: Chem Sci. 2015 Sep 15;6(12):7150–5. doi: 10.1039/c5sc03174e (PMC5952552; doi:10.1039/c5sc03174e)
Supplement: Supplementary file 1 [file SC-006-C5SC03174E-s001.pdf]

Electronic Supplementary Material (ESI) for Chemical Science.
This journal is © The Royal Society of Chemistry 2015

SUPPORTING INFORMATION

Reversible [4+2] Cycloaddition Reaction of 1,3,2,5-Diazadiborinine with Ethylene

Di Wu,[†] Rakesh Ganguly,[‡] Yongxin Li,[‡] Sin Ni Hoo,[†] Hajime Hirao*[,†] and Rei Kinjo*[,†]

† Division of Chemistry and Biological Chemistry, School of Physical and Mathematical Sciences, and ‡ NTU-CBC Crystallography Facility, Nanyang Technological University, Singapore 637371, Singapore

CONTENTS

1. Synthesis, physical and spectroscopic data

2. Kinetic study

3. Crystal Structure Determination

4. DFT Calculation

5. References

6. NMR spectra

1. Synthesis, physical and spectroscopic data for all new compounds

General considerations: All reactions were performed under an atmosphere of dry argon using standard Schlenk or dry box techniques; solvents were dried over Na metal, K metal or CaH_2., and distilled under nitrogen. Reagents were of analytical grade, obtained from commercial suppliers and used without further purification. ^{1}H, ^{13}C, ^{11}B and ^{19}F NMR spectra were recorded on a Bruker AVIII 400MHz or Bruker Avance 500MHz AV500, spectrometers at 298 K. NMR multiplicities are abbreviated as follows: s = singlet, d = doublet, m = multiplet. Coupling constants *J* are given in Hz. In the ^{13}C NMR spectra of compounds **2**, **4a-4e**, **6**, signals for the carbon atoms directly bonding to the boron atom could not be observed, presumable due to the coupling with the boron atom. Electrospray ionization (ESI) mass spectra were obtained at the Mass Spectrometry Laboratory at the Division of Chemistry and Biological Chemistry, Nanyang Technological University. Melting points were measured with OptiMelt (Stanford Research System).

Compound 2: A C_6D_6 (0.35 mL) solution of 1,3,2,5-diazadiborinine **1** (0.010 g, 0.027 mmol) was added to a J-Young NMR tube. The sample was degassed using a freeze-pump-thaw method. Ethylene (1 bar) was introduced into the NMR tube at room temperature, and the reaction was monitored by NMR spectroscopy. After 3 hour, all volatiles were removed under vacuum, which afforded a white powder of **2** (97 % yield).

Mp: 156 °C. ^{1}H NMR (500 MHz, C_6D_6): δ = 0.51 (s, 6 H), 0.91 (s, 6 H), 1.05-1.08 (m, 2 H), 1.25-1.28 (m, 2 H), 3.35 (d, *J* = 8.5 Hz, 2 H), 3.41 (d, *J* = 9.0 Hz, 2 H), 7.25-8.14 (m, 10 H); ^{13}C NMR (125 MHz, C_6D_6): δ = 26.1, 28.0, 65.3, 83.3, 126.0, 127.5, 127.8, 134.8; ^{13}C NMR (DEPT-135, 125 MHz, C_6D_6): δ = 26.1, 28.0, 126.0, 127.5, 127.8, 128.0, 128.4, 134.8; ^{11}B NMR (76.8 MHz, C_6D_6): δ = –16.0 (s), 1.6 (s). HRMS (ESI): *m/z* calcd for $C_{24}H_{31}B_2N_2O_2$: 401.2572 $[(M+H)]^+$; found: 401.2581.

Compound 4a: Styrene **3a** (6.2 μL, 0.054 mmol) was added into a C_6D_6 (0.6 mL) solution of **1** (0.010 g, 0.027 mmol) in a J-Young NMR tube at room temperature. The reaction was monitored by NMR spectroscopy. After 12 hours, all volatiles were removed under vacuum. The residue was washed with hexane and dried under vacuum to afford a colorless solid of **4a** (90 %).

Mp: 132 °C. ^{1}H NMR (400 MHz, C_6D_6) δ = 0.57 (s, 3 H), 0.59 (s, 3 H), 0.91 (s, 3 H), 1.1 (s, 3 H), 1.29 (dd, *J* = 5.0 Hz, *J* = 13.6 Hz, 1 H), 1.84 (dd, *J* = 10 Hz, *J* = 13.6 Hz, 1 H), 2.72 (dd, *J* = 5.0 Hz, *J* = 10 Hz, 1 H), 3.37 (d, *J* = 8.8 Hz, 1 H), 3.48 (d, *J* = 8.8 Hz, 1 H), 3.61 (d, *J* = 8.8 Hz, 1 H), 3.70 (d, *J* = 8.8 Hz, 1 H), 6.99-8.02 (m, 15 H); ^{13}C NMR (100 MHz, C_6D_6): δ = 25.4, 25.8, 27.8, 29.0, 65.0, 65.4, 83.4, 83.8, 123.9, 125.5, 127.1, 127.9, 128.2, 135.1, 151.9; ^{13}C NMR (DEPT-135, 100 MHz, C_6D_6): δ = 25.4, 25.8, 27.8, 29.0, 123.9, 125.5, 127.1 (*C*H x2), 127.9 (*C*H x5), 128.2 (*C*H x3), 135.1 (*C*H x3); ^{11}B NMR (76.8 MHz, C_6D_6): δ = –14.3 (s), 0.8 (s); HRMS (ESI): *m/z*

calcd for $C_{30}H_{35}B_2N_2O_2$: 477.2885 [(*M*+*H*)]$^+$; found: 477.2899.

Compound 4b: By following the procedure for the synthesis of **4a**, the reaction of **1** and **3b** afforded **4b** as a colorless solid (83 %).

Mp: 116 ^{o}C. ^{1}H NMR (400 MHz, C_6D_6): δ = 0.56 (s, 3 H), 0.60 (s, 3 H), 0.89 (s, 3 H), 1.07 (s, 3H), 1.28 (dd, *J* = 5.0 Hz, *J* = 13.5 Hz, 1 H), 1.84 (dd, *J* = 9.9 Hz, *J* = 13.5 Hz, 1 H), 2.13(s, 3 H), 2.73 (dd, *J* = 5.0 Hz, *J* = 9.9 Hz, 1 H), 3.34 (d, *J* = 8.8 Hz, 1 H), 3.45 (d, *J* = 8.8 Hz, 1 H), 3.61 (d, *J* = 8.8 Hz, 1 H), 3.72 (d, *J* = 8.8 Hz, 1 H), 6.95-8.04 (m, 14 H); ^{13}C NMR (100 MHz, C_6D_6): δ = 21.1, 25.5, 25.8, 27.8, 29.0, 65.0, 65.4, 83.4, 83.8, 125.5, 127.1, 128.7, 132.6, 135.2, 148.7; ^{13}C NMR (DEPT-135, 100 MHz, C_6D_6): δ = 21.1, 25.5, 25.8, 27.8, 29.0, 125.5, 127.1 (*C*H x2), 127.9, 128.1 (*C*H x3), 128.4, 128.7 (*C*H x3), 135.2 (*C*H x3); ^{11}B NMR (76.8 MHz, C_6D_6): δ = –14.3 (s), 0.9 (s) ; HRMS (ESI): *m/z* calcd for $C_{31}H_{37}B_2N_2O_2$: 491.3041 [(*M*+*H*)]$^+$; found: 491.3050.

Compound 4c: By following the procedure for the synthesis of **4a**, the reaction of **1** and **3c** afforded **4c** as a colorless solid (85 %).

Mp: 117 ^{o}C. ^{1}H NMR (400 MHz, C_6D_6): δ = 0.57 (s, 3 H), 0.58 (s, 3 H), 0.89 (s, 3 H), 1.06 (s, 3 H), 1.25 (dd, *J* = 5.0 Hz, *J* = 13.6 Hz, 1 H), 1.85 (dd, *J* = 10 Hz, *J* = 13.6 Hz, 1 H), 2.73 (dd, *J* = 5.0 Hz, *J* = 10 Hz, 1 H), 3.32 (s, 3 H), 3.34 (d, *J* = 8.8 Hz, 1 H), 3.46 (d, *J* = 8.8 Hz, 1 H), 3.60 (d, *J* = 8.8 Hz, 1 H), 3.68 (d, *J* = 8.8 Hz, 1 H), 6.76-8.05 (m, 14 H); ^{13}C NMR (100 MHz, C_6D_6): δ = 25.5, 25.8, 27.8, 28.9, 54.7, 65.0, 65.4, 83.4, 83.8, 113.6, 125.6, 127.1, 127.9, 128.9, 135.2, 143.6, 157.0; ^{13}C NMR (DEPT-135, 100 MHz, C_6D_6): δ = 25.5, 25.8, 27.8, 28.9, 54.7, 113.6 (*C*H x3), 125.6, 127.1 (*C*H x2), 127.9, 128.4, 128.9 (*C*H x3), 135.2 (*C*H x3); ^{11}B NMR (76.8 MHz, C_6D_6): δ = –14.3 (s), 1.1 (s); HRMS (ESI): *m/z* calcd for $C_{31}H_{37}B_2N_2O_3$: 507.2990 [(*M*+*H*)]$^+$; found: 507.3000.

Compound 4d: By following the procedure for the synthesis of **4a**, the reaction of **1** and **3d** afforded **4d** as a colorless solid (89 %).

Mp: 273 ^{o}C. ^{1}H NMR (400 MHz, C_6D_6): δ = 0.51 (s, 3 H), 0.53 (s, 3 H), 0.87 (s, 3 H), 1.04 (s, 3 H), 1.13 (dd, *J* = 5.0 Hz, *J* = 13.6 Hz, 1 H), 1.73 (dd, *J* = 9.8 Hz, *J* = 13.6 Hz, 1 H), 2.56 (dd, *J* = 5.0 Hz, *J* = 9.8 Hz, 1 H), 3.32 (d, *J* = 8.8 Hz, 1 H), 3.44 (d, *J* = 8.8 Hz, 1 H), 3.56 (s, 2 H), 6.82-7.95 (m, 14 H); ^{13}C NMR (100 MHz, C_6D_6): δ = 25.4, 25.8, 27.8, 28.9, 65.0, 65.5, 83.4, 83.8, 117.4, 125.8, 127.2, 128.0, 129.8, 130.8, 135.0, 151.0; ^{13}C NMR (DEPT-135, 100 MHz, C_6D_6): δ = 25.4, 25.8, 27.8, 28.9, 125.8, 127.2 (*C*H x2), 128.0, 128.4, 129.8 (*C*H x3), 130.8 (*C*H x3), 135.0 (*C*H x3); ^{11}B NMR (76.8 MHz, C_6D_6): δ = –14.4 (s), 1.0 (s); HRMS (ESI): *m/z* calcd for $C_{30}H_{34}B_2N_2O_2Br$: 555.1990 [(*M*+*H*)]$^+$; found: 555.2003.

Compound 4e: By following the procedure for the synthesis of **4a**, the reaction of **1** and **3e** afforded **4e** as a colorless solid (86 %).

Mp: 150 ^{o}C. ^{1}H NMR (400 MHz, C_6D_6): δ = 0.53 (s, 3 H), 0.54 (s, 3 H) 0.88 (s, 3 H), 1.04 (s, 3 H), 1.16 (dd, J = 4.9 Hz, J = 13.7 Hz, 1 H), 1.73 (dd, J = 9.8 Hz, J = 13.7 Hz, 1 H), 2.60 (dd, J = 4.9 Hz, J = 9.8 Hz, 1 H), 3.34 (d, J = 8.8 Hz, 1 H), 3.46 (d, J = 8.8 Hz, 1 H), 3.56 (d, J = 9.0 Hz, 1 H), 3.59 (d, J = 9.0 Hz, 1 H), 6.97-7.90 (m, 14 H); ^{13}C NMR (100 MHz, C_6D_6): δ = 25.4, 25.8, 27.8, 29.0, 65.1, 65.5, 83.5, 83.9, 124.67, 124.70, 124.74, 125.9, 127.2, 134.9, 156.7; ^{13}C NMR (DEPT-135, 100 MHz, C_6D_6): δ = 25.4, 25.8, 27.8, 29.0, 124.67, 124.70, 124.74, 125.9, 127.2, 128.1, 128.4, 134.9; ^{11}B NMR (76.8 MHz, C_6D_6): δ = –14.4 (s), 1.0 (s); ^{19}F NMR (225.6 MHz, C_6D_6): δ = –61.4; HRMS (ESI): *m/z* calcd for $C_{31}H_{34}B_2N_2O_2F_3$: 545.2758 [(M+H)]$^+$; found: 545.2769.

Compound 6: Norbornene **5** (0.006 g, 0.065 mmol) was added into a C_6D_6 (0.5 mL) solution of **1** (0.020 g, 0.054 mmol) in a J-Young NMR tube. The reaction was monitored by NMR spectroscopy. After heating at 90 ^{o}C for 12 hours, the solvent was removed under vacuum. The resulting residue was recrystallized from benzene/hexane to afford colorless crystals of **6** (83 %).

Mp: 250 ^{o}C. ^{1}H NMR (400 MHz, C_6D_6): δ = 0.49 (s, 3 H), 0.55 (s, 3 H), 0.75 (d, J = 9.6 Hz, 1 H) 0.82 (s, 3 H), 0.90 (d, J = 9.6 Hz, 1 H), 1.19 (s, 3 H), 1.30-2.65 (m, 8 H), 3.32 (d, J = 8.8 Hz, 1 H), 3.37 (d, J = 8.8 Hz, 1 H), 3.52 (d, J = 8.8 Hz, 1 H), 3.64 (d, J = 8.8 Hz, 1 H), 7.24-8.27 (m, 10 H); ^{13}C NMR (100 MHz, C_6D_6): δ = 23.9, 25.8, 28.2, 30.4, 34.3, 34.5, 35.3, 40.3, 41.2, 64.3, 64.9, 83.8, 84.7, 125.7, 127.5, 127.7, 135.2; ^{13}C NMR (DEPT-135, 100 MHz, C_6D_6): δ = 23.9, 25.8, 28.2, 30.4, 40.3, 41.2, 125.7, 127.5 (*C*H x3), 127.7 (*C*H x2), 128.4 (*C*H x2), 135.2 (*C*H x3); ^{11}B NMR (76.8 MHz, C_6D_6): δ = –15.8 (s), 1.3 (s); HRMS (ESI): *m/z* calcd for $C_{29}H_{37}B_2N_2O_2$: 467.3041 [(M+H)]$^+$; found: 467.3051.

2. Kinetic study

General procedure for the reaction of 1 and 3a at various temperatures: **1** (0.014 mmol), **3a** (0.067 mmol), the internal standard 1,4-di-tert-butylbenzene (0.013 mmol), and THF-d_8 (0.5 mL) were loaded into a dried J-Young-Tube under argon atmosphere. The tube was sealed and heated over a temperature range from 27 ^{o}C to 65 ^{o}C. The reaction was monitored by ^{1}H NMR spectroscopy. Based on the integration of **1**, the concentration of **1** was plotted against time which follows first-order kinetics (Figure S2-1). The Eyring plot (Figure S2-2) was obtained based on the rate at each temperature and plotted against inverse of temperature.

**27 ^{o}C**		
**Time / s**	**[1] / M**	**Ln [1]**
0	0.018072232	-4.013379
840	0.017598936	-4.039917
1260	0.016425156	-4.108941
1560	0.015763341	-4.150068
1800	0.015206609	-4.186025
2160	0.014499609	-4.233634
2460	0.013962633	-4.271371
2760	0.013336756	-4.317231
3060	0.012834038	-4.355654
3360	0.012201016	-4.406236
3660	0.011724778	-4.446051
3960	0.011298560	-4.48308
4260	0.010826526	-4.525756
4560	0.010396735	-4.566263
4860	0.009998049	-4.605365
5160	0.009540936	-4.652164
5460	0.009249855	-4.683147
5760	0.008851800	-4.727134

**40 ^{o}C**		
**Time / s**	**[1] / M**	**Ln [1]**
0	0.018023893	-4.016056994
360	0.016039079	-4.132727076

480	0.015125905	-4.191346431
600	0.014486158	-4.234561718
720	0.013978395	-4.270242344
840	0.013191531	-4.328180234
960	0.012627024	-4.371916039
1080	0.012101817	-4.414399672
1200	0.011506625	-4.464832323
1320	0.011011683	-4.508798524
1440	0.010504550	-4.555946751
1560	0.010070346	-4.598160213
1680	0.009620800	-4.643827910
1800	0.009386254	-4.668509054
1920	0.009034435	-4.706711947
2040	0.008518055	-4.765567251
2160	0.008284980	-4.793311022
2280	0.007989276	-4.829655178
2400	0.007527329	-4.889214970
2520	0.007282906	-4.922225389
2640	0.006979215	-4.964818880

55 ^{o}C		
**Time / s**	**[1] / M**	**Ln [1]**
0	0.018023893	-4.016056994
540	0.013171565	-4.329694914
660	0.011502422	-4.465197686
780	0.010372356	-4.568611137
900	0.009239347	-4.684284067
1020	0.008317136	-4.789437354
1140	0.007692731	-4.867479487
1260	0.006948741	-4.969194859
1380	0.006398524	-5.051687914
1500	0.005916612	-5.129991291
1620	0.005534529	-5.196748811
1740	0.005232520	-5.252862377
1860	0.004831101	-5.332680852
1980	0.004493363	-5.405153786
2100	0.004323549	-5.443678763
2220	0.004202072	-5.472177463

65 ^{o}C		
**Time / s**	**[1] / M**	**Ln [1]**
0	0.018023893	-4.016056994
180	0.011479303	-4.467209575
260	0.009933948	-4.611797314
340	0.008859786	-4.726232668
420	0.007720893	-4.863825270
500	0.007096488	-4.948155312
580	0.006403148	-5.050965561
660	0.005948137	-5.124677218
740	0.005393928	-5.222481495
820	0.005083722	-5.281711707

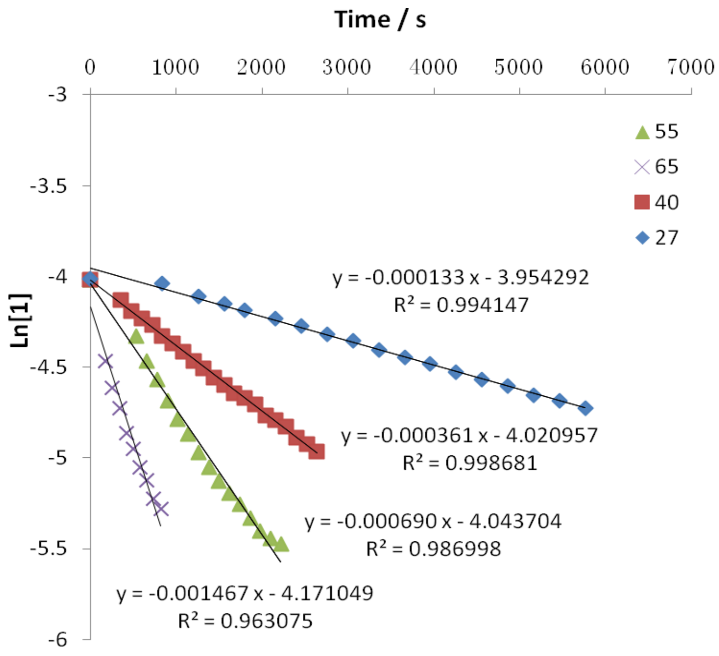

Figure S2-1. Plots of ln [**1**] against time to determine rate constant (k) at various temperatures.

T/K	$1/(T/K^{-1})$	k/s^{-1}	$(k/s^{-1})/(T/K)$	Ln(k/T)
300.15	0.003331667	0.000133	4.43112E-07	-14.62944378
313.15	0.003193358	0.000361	1.1528E-06	-13.67331491
328.15	0.003047387	0.000690	2.1027E-06	-13.07228978
338.15	0.002957267	0.001467	4.33831E-06	-12.34802536

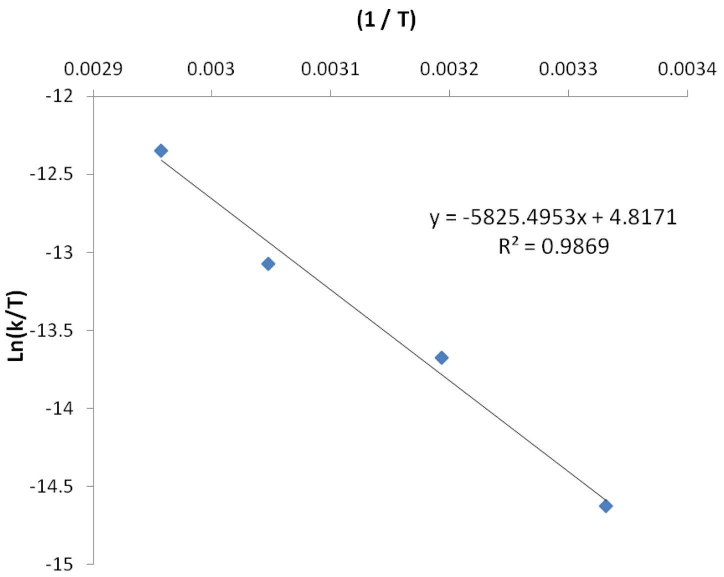

$\Delta H^{\ddagger}$ = 11.6 ± 0.9 kcal/mol

$\Delta S^{\ddagger}$ = –37.6 ± 3.0 e.u.

$\Delta G^{\ddagger}$ = 22.8 ± 1.8 Kcal/mol

Figure S2-2. Eyring plot for the cycloaddition reaction between **1** and **3a**

General procedure for the Hammett Plot: Compound **1** (0.014 mmol), styrene (0.067 mmol), internal standard 1,4-di-tert-butylbenzene (0.013 mmol), and THF-d_8 (0.5 mL) were loaded in a dried J-Young-Tube under argon atmosphere. The tube was sealed and the reaction was monitored by NMR spectroscopy at room temperature. The rate constant (k) for each substrate **3a-e** was obtained from the plot of substrate concentration against time. The Hammett plot is obtained by plotting k against substituent coefficient σ (Figure S2-3).[1]

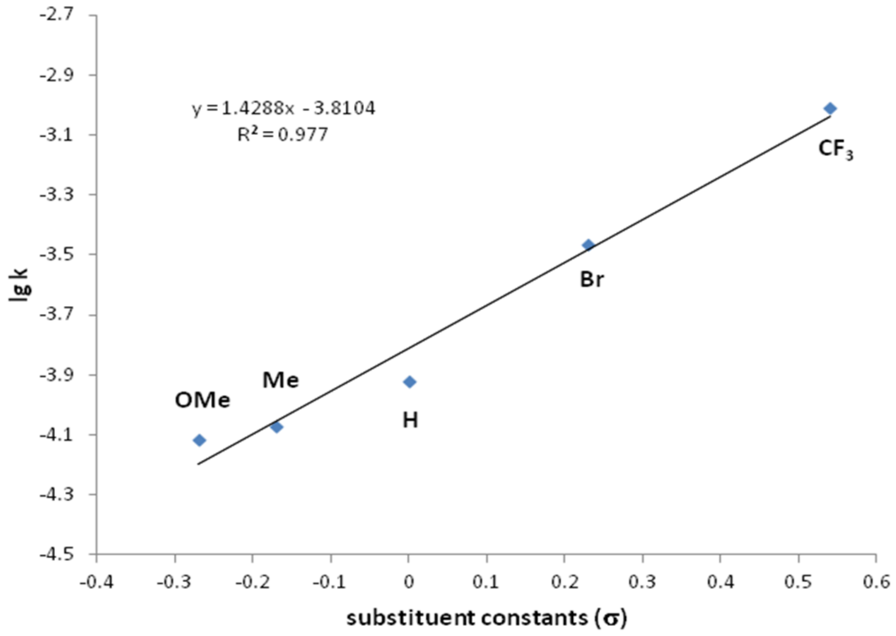

para-substituents	Substituent constant	lg k
OMe (**3c**)	-0.27	-4.113509275
Me (**3b**)	-0.17	-4.070581074
H (**3a**)	0.00	-3.920818754
Br (**3d**)	0.23	-3.465973894
CF_3 (**3e**)	0.54	-3.009661145

Figure S2-3. Hammett plots with ρ = + 1.43: substitution at the para-position on phenyl group.

General procedure to estimate equilibrium constants (K_{4-1}) at room temperature: Compound **4** (0.015 mmol), internal standard 1,4-di-tert-butylbenzene (0.015 mmol) and C_6D_6 (0.6 mL) were loaded in a dried J-Young-Tube under argon atmosphere. The tube was sealed and the reaction was monitored by NMR spectroscopy at room temperature.

Table S2-1. Summary of reaction time (h) vs the conversion (%) of **4** to **1** and **3** under the reaction condition.

	12h	36h	60h	84h	108h
**4a**	7.60%	14.60%	16.90%	18.00%	18.00%
**4b**	12.00%	18.40%	21.00%	21.60%	21.60%
**4c**	14.70%	21.60%	24.20%	25.10%	25.10%
**4d**	5.30%	6.70%	7.10%	7.10%	7.10%
**4e**	4.00%	4.30%	4.60%	4.60%	4.60%

Table S2-2. Summary of the concentration (M) of [**4**] and [**1**] (= [**3**]) in the equilibrium states.

Concentrations of **4** and **1** in equilibrium			
para-substituents	[**4**]	[**1**]	equilibrium constants (K_{4-1}) at r.t.
**H**	0.01764	0.00392	1.148×10^3
**Me**	0.02050	0.00584	0.601×10^3
**OMe**	0.01890	0.00643	0.457×10^3
**Br**	0.01993	0.00151	8.741×10^3
**CF_3**	0.01931	0.00098	2.011×10^4

General procedure for retro Diels-alder reaction of 4a-e at high temperature: Compound **4** (0.006 mmol) and C_6D_6 (0.4 mL) were loaded in a dried J-Young-Tube under argon atmosphere. The tube was sealed and heated over a temperature range from 110 ^oC to 150 ^oC. The reaction was monitored by NMR spectroscopy.

Table S2-3. Summary of the reaction time *vs* the conversion (%) of **4** to **1** and **3** under the reaction temperature (110 ~ 150 ^oC).

T	**4a**	**4b**	**4c**	**4d**	**4e**
110 ^oC	25 min (100%)	15 min (100%)	13 min (100%)	30 min (73%)	30 min (60%)
130 ^oC				60 min (85%)	60 min (79%)
150 ^oC				120 min (87%)	120 min (80%)
150 ^oC				12 h (87%)	12 h (80%)

3. Crystal Structure Determination of Compounds 2, 4a, 4b, 4d, and 6

X-ray data collection and structural refinement. Intensity data for compounds **2**, **4a**, **4b**, **4d** and **6** were collected using a Bruker APEX II diffractometer. The crystals of **2**, **4a**, **4d** and **6** were measured at 153(2) K and the crystal of **4b** was measured at 103(2). The structure was solved by direct phase determination (SHELXS-97)[2] and refined for all data by full-matrix least squares methods on F^2.[3] All non-hydrogen atoms were subjected to anisotropic refinement. The hydrogen atoms were generated geometrically and allowed to ride in their respective parent atoms; they were assigned appropriate isotropic thermal parameters and included in the structure-factor calculations. CCDC:1418724-1418728 contains the supplementary crystallographic data for this paper. The data can be obtained free of charge from the Cambridge Crystallography Data Center via www.ccdc.cam.ac.uk/data_request/cif.

Table S3-1. Summary of Data Collection and Structure Refinement.

	**2·(C$_6$D$_6$)$_{1/2}$**	**4a**	**4b**
Formula	$C_{27}H_{30}B_2D_3N_2O_2$	$C_{30}H_{34}B_2N_2O_2$	$C_{31}H_{36}B_2N_2O_2$
Fw	422.19	476.21	490.24
cryst syst	triclinic	triclinic	monoclinic
space group	*P-1*	*P-1*	*P 1 21/n 1*
Size (mm^3)	0.200 x 0.180 x 0.020	0.040 x 0.100 x 0.180	0.360 x 0.400 x 0.420
T, K	153(2)	153(2)	103(2)
a, Å	6.2435(5)	12.0335(18)	9.498(2)
b, Å	13.8921(9)	13.357(2)	9.090(2)
c, Å	14.2338(10)	17.045(3)	30.647(7)
α, deg	100.533(4) °	73.663(4)°	90°
β, deg	92.139(5)°	81.968(4)°	94.119(3)°
γ, deg	100.164(5) °	79.686(4)°	90°
V, A^3	1191.63(15)	2575.0(7)	2639.1(11)
Z	2	4	4
d_{calcd}g·cm^{-3}	1.232	1.228	1.234
μ, mm^{-1}	0.075	0.075	0.075
Refl collected	4727	9199	4811
T_{min}/T_{max}	0.79/1.00	0.9870/0.9970	0.9690/0.9730
N$_{measd}$	4727	9199	4811
[R$_{int}$]	0.1367	0.0814	0.0862
R [I>2sigma(I)]	0.0771	0.0858	0.0595
R_w[I>2sigma(I)]	0.1726	0.2381	0.1579
GOF	0.928	1.046	1.013
Largest diff peak/hole[e·Å^{-3}]	0.377/-0.238	0.555/-0.376	0.280/-0.365

	**4d**	**$6\cdot(C_6H_6)_{1/2}$**
Formula	$C_{30}H_{33}B_2BrN_2O_2$	$C_{32}H_{39}B_2N_2O_2$
Fw	555.11	505.27
cryst syst	monoclinic	monoclinic
space group	*P 1 21/n 1*	*P 1 21/n 1*
Size (mm^3)	0.110 x 0.120 x 0.420	0.180 x 0.220 x 0.300
T, K	153(2)	153(2)
a, Å	9.5066(6)	8.8862(4)
b, Å	9.2480(5)	21.9850(11)
c, Å	30.632(2)	14.0495(7)
α, deg	90°	90°
β, deg	94.399(3)^o	96.2349(19)°
γ, deg	90°	90°
V, A^3	2685.1(3)	2728.5(2)
Z	4	4
d_{calcd} g·cm^{-3}	1.373	1.230
μ, mm^{-1}	1.562	0.075
Refl collected	17487	44526
T_{min}/T_{max}	0.5600/0.8470	0.9780/0.9870
N_{measd}	7720	6540
[R_{int}]	0.0636	0.0827
R [I>2sigma(I)]	0.0583	0.0502
R_w[I>2sigma(I)]	0.1268	0.1777
GOF	0.986	1.093
Largest diff peak/hole[e·Å^{-3}]	0.442 /-0.702	0.456/-0.504

4. DFT Calculation

Method

All density functional theory (DFT) calculations were performed using Gaussian 09.[4] The M06-2X functional was used in conjunction with the 6-31G(d) basis set (B1) for geometry optimization and frequency calculations.[5,6] The M06-2X/B1 calculations, which yielded free energy correction values (G_{corr}), were followed by single-point energy calculations at the M06-2X(SCRF)/B2//M06-2X/B1 level, where SCRF stands for a self-consistent reaction field model called IEFPCM to describe the solvent effect of benzene implicitly,[7] and B2 is the def2-TZVP basis set.[8] The energy obtained from the B2 calculation is referred to as E(B2). The following quantity G was used to evaluate the relative stability of different species.

$$G = E(B2) + G_{corr}$$

	E(B1) [au]	ZPE [au]	G_{corr} [au]	E(B2) [kcal/mol]	ΔG [kcal/mol]
**1**	-1163.275382	0.456768	0.402862	-1163.710364	
**3a**	-309.499518	0.134589	0.104686	-309.618074	
**1** + **3a**	-1472.774900	0.591357	0.507548	-1473.328438	0.0
**RCA**	-1472.797741	0.594082	0.529794	-1473.344901	3.6
**RCB**	-1472.797505	0.593558	0.530796	-1473.344503	4.5
**TSA**	-1472.774610	0.593314	0.532590	-1473.319729	21.2
**TSB**	-1472.772560	0.594128	0.534396	-1473.317565	23.7
**4a(A)**	-1472.819330	0.596051	0.535589	-1473.361109	-2.9
**4a(B)**	-1472.815660	0.596568	0.537085	-1473.357273	0.4

Figure S4-1. Key frontier orbitals of **1** (a) and **3a** (b) (M06/6-31G*). Orbital energies (in hartrees) are shown in parentheses. Hydrogen atoms are omitted for clarity.

(a) **1**

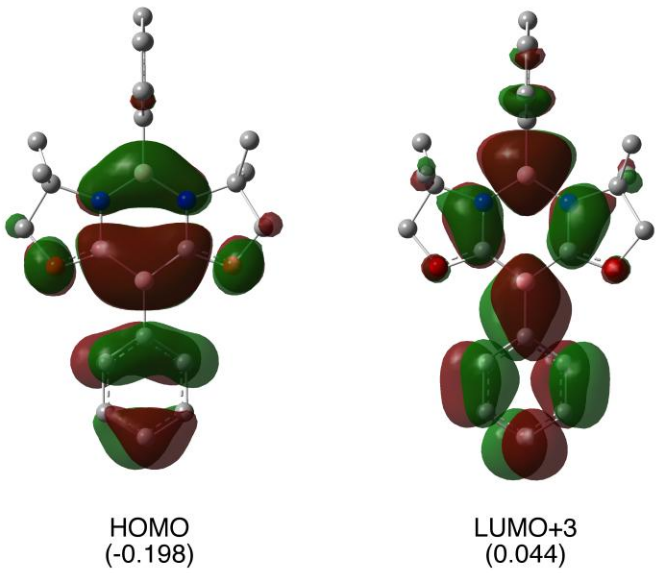

(b) **3a**

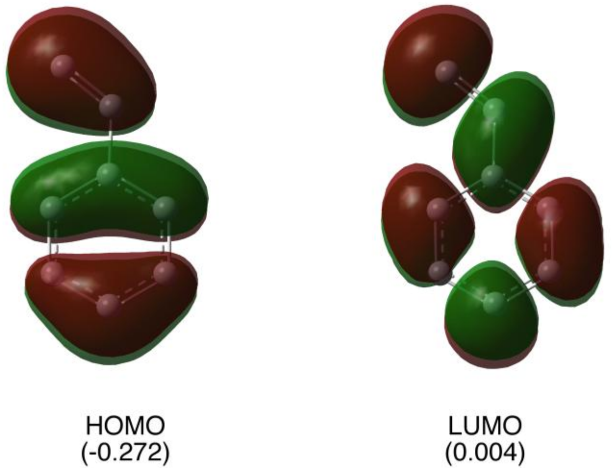

Figure S4-2. Key Mulliken charges of **1** (a) and **3a** (b) (M06/6-31G*). Hydrogen atoms are omitted for clarity.

(a) **1**

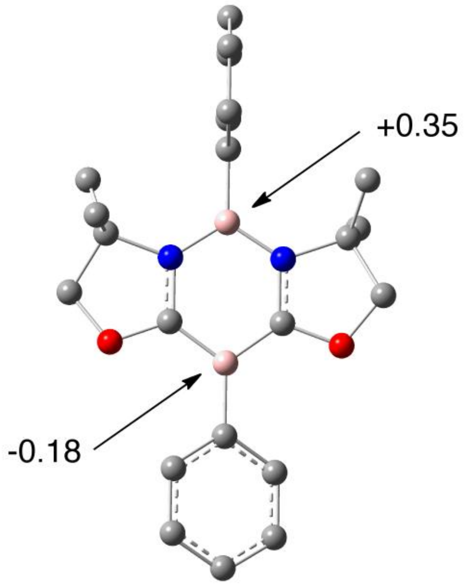

(b) **3a**

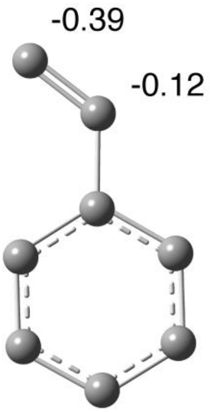

XYZ Coordinates of Optimized Geometries

=== **1** ===

B	1.877167	-0.000073	0.000185
C	3.449107	-0.000109	0.000095
C	1.004437	-1.208402	-0.092896
C	1.004514	1.208298	0.093307
C	4.186191	1.140717	-0.362674
C	4.186196	-1.140966	0.362761
N	-0.374188	-1.205567	-0.101229
O	1.462053	-2.484426	-0.173649
N	-0.374104	1.205529	0.101674
O	1.462188	2.484301	0.174019
H	3.656339	2.043933	-0.650615
C	5.577979	1.144464	-0.364141
H	3.656348	-2.044161	0.650781
C	5.577986	-1.144773	0.364021
B	-1.144652	0.000016	0.000232
C	-0.866196	-2.611911	-0.065929
C	0.389221	-3.321376	-0.579880
C	-0.866061	2.611876	0.066386
C	0.389404	3.321308	0.580268
H	6.115752	2.044268	-0.651580
C	6.282637	-0.000169	-0.000115
H	6.115763	-2.044600	0.651380
C	-2.723277	0.000116	0.000061
C	-1.158976	-3.008354	1.381864
C	-2.055641	-2.897651	-0.975215
H	0.530477	-4.313017	-0.145161
H	0.382978	-3.393338	-1.676060
C	-1.158931	3.008293	-1.381395
C	-2.055469	2.897590	0.975727
H	0.530680	4.312933	0.145519
H	0.383208	3.393299	1.676446
H	7.369063	-0.000193	-0.000198
C	-3.450083	-0.148305	1.189584
C	-3.449685	0.148697	-1.189685
H	-2.140058	-3.982310	-1.108878
H	-2.993864	-2.535338	-0.551254
H	-0.271942	-2.857428	2.004994
H	-1.449609	-4.063092	1.431882
H	-1.981012	-2.407762	1.780763
H	-1.913438	-2.439703	-1.958716
H	-2.139967	3.982250	1.109331
H	-2.993684	2.535170	0.551838
H	-0.271927	2.857393	-2.004571
H	-1.449602	4.063020	-1.431405
H	-1.980969	2.407668	-1.780242
H	-1.913174	2.439710	1.959246
H	-2.917669	-0.250389	2.133282
C	-4.843088	-0.150571	1.194292
H	-2.916952	0.250760	-2.133203
C	-4.842685	0.151125	-1.194847
H	-5.381748	-0.266280	2.130206
C	-5.543005	0.000304	-0.000393
H	-5.381029	0.266929	-2.130930
H	-6.628801	0.000370	-0.000566
H	0.490188	-2.746430	2.984114

=== **3a** ===

C	-0.286889	-0.065585	-4.038686
C	0.812184	-0.420798	-3.372599
H	-0.832190	0.833334	-3.772703
H	-0.686477	-0.646501	-4.864672
H	1.149333	0.214195	-2.554011
C	1.653045	-1.607416	-3.615970
C	2.770708	-1.819480	-2.801586
C	1.384535	-2.538246	-4.628785
C	3.597296	-2.923193	-2.986224
H	2.991218	-1.105239	-2.011860
C	2.207416	-3.640771	-4.815418
H	0.524788	-2.399052	-5.277359
C	3.317804	-3.839143	-3.995149
H	4.459004	-3.066875	-2.341310
H	1.983115	-4.350993	-5.605737
H	3.959288	-4.702234	-4.144558

=== **RCA** ===

B	2.020485	0.271748	0.569896
C	3.591367	0.355722	0.631918
C	1.229609	-0.993150	0.417843
C	1.076893	1.427922	0.544756
C	4.271645	1.566386	0.404142
C	4.390490	-0.779453	0.867056
N	-0.130500	-1.067835	0.246118
O	1.764678	-2.234956	0.424064
N	-0.293566	1.342136	0.401740
O	1.447270	2.732870	0.637377
H	3.699461	2.468234	0.210857
C	5.661219	1.642999	0.415934
H	3.913108	-1.737099	1.048235
C	5.781246	-0.709044	0.878712
B	-0.976673	0.092334	0.227954
C	-0.550815	-2.498193	0.214159
C	0.802014	-3.152699	-0.085003
C	-0.856516	2.714020	0.261194
C	0.288292	3.513619	0.887445
H	6.149342	2.597287	0.235847
C	6.426195	0.504064	0.653267
H	6.363601	-1.606838	1.070176
C	-2.544605	0.005402	0.068198
C	-1.062430	-2.908852	1.595001
C	-1.557225	-2.825532	-0.883681
H	0.932047	-4.110696	0.423204
H	0.963396	-3.273893	-1.162748
C	-1.003065	3.042972	-1.225054
C	-2.157015	2.959844	1.017389
H	0.421685	4.498753	0.435621
H	0.150523	3.617984	1.972313
H	7.511002	0.561281	0.662697
C	-3.373413	-0.127954	1.190976
C	-3.159491	0.079746	-1.189672
H	-1.533942	-3.905032	-1.072403
H	-2.575210	-2.551117	-0.601208
H	-0.295766	-2.729274	2.355502
H	-1.315794	-3.974420	1.597318
H	-1.961722	-2.343917	1.854086
H	-1.291998	-2.306466	-1.810309
H	-2.309828	4.041858	1.104160
H	-3.020118	2.536384	0.500960
H	-0.041224	2.932087	-1.738011
H	-1.353346	4.072667	-1.354106
H	-1.733162	2.370732	-1.685120
H	-2.106688	2.536769	2.025028
H	-2.927513	-0.175313	2.182792
C	-4.759753	-0.181173	1.068298
H	-2.538651	0.155360	-2.080688
C	-4.545457	0.032170	-1.321112
H	-5.379659	-0.281622	1.954447

C	-5.349182	-0.098150	-0.190863
H	-4.998618	0.093010	-2.306350
H	-6.429782	-0.136158	-0.291125
C	-0.386344	0.365272	-3.787582
C	0.560237	-0.052885	-2.943136
H	-0.889081	1.315929	-3.639824
H	-0.667667	-0.203885	-4.669775
H	0.845254	0.573400	-2.095180
C	1.321609	-1.311915	-3.066289
C	2.570194	-1.420447	-2.441338
C	0.831900	-2.413122	-3.783611
C	3.308906	-2.597132	-2.526445
H	2.959302	-0.582404	-1.868374
C	1.571410	-3.586564	-3.873711
H	-0.146422	-2.354875	-4.253946
C	2.812242	-3.683532	-3.241697
H	4.266756	-2.659043	-2.018623
H	1.175026	-4.432624	-4.427570
H	3.383365	-4.605085	-3.302988

=== **RCB** ===

B	1.539419	-0.271632	-0.049346
C	3.052796	-0.508142	-0.404813
C	0.505348	-1.350754	0.061319
C	0.875411	1.048363	0.147345
C	3.836051	0.517189	-0.964895
C	3.676590	-1.758222	-0.235201
N	-0.842830	-1.150747	0.218927
O	0.780427	-2.672338	0.019504
N	-0.475486	1.240638	0.360080
O	1.514634	2.249054	0.137983
H	3.393978	1.497924	-1.114913
C	5.160966	0.310186	-1.336559
H	3.111446	-2.578082	0.197951
C	5.001918	-1.972314	-0.606017
B	-1.417601	0.158176	0.371618
C	-1.532752	-2.468016	0.334937
C	-0.430408	-3.386076	-0.207603
C	-0.760216	2.703367	0.387038
C	0.645945	3.217797	0.706104
H	5.733702	1.126162	-1.769629
C	5.752163	-0.938650	-1.160954
H	5.452332	-2.950489	-0.457113
C	-2.970187	0.392535	0.541396
C	-1.819772	-2.766728	1.806222
C	-2.785825	-2.587103	-0.525994
H	-0.375301	-4.341482	0.319001
H	-0.542781	-3.557753	-1.284281
C	-1.200974	3.164238	-1.002472
C	-1.749570	3.143131	1.459564
H	0.859085	4.190666	0.258169
H	0.813918	3.262045	1.790963
H	6.785710	-1.104300	-1.451662
C	-3.580997	0.402577	1.803160
C	-3.786505	0.610915	-0.577477
H	-3.030976	-3.648922	-0.644300
H	-3.644219	-2.087717	-0.073075
H	-0.894197	-2.733962	2.389771
H	-2.261435	-3.764038	1.905379
H	-2.525972	-2.039677	2.215146
H	-2.603852	-2.161300	-1.518301
H	-1.650555	4.225280	1.603473
H	-2.782320	2.932263	1.176568
H	-0.435348	2.920123	-1.746122
H	-1.362183	4.247504	-1.005910
H	-2.138731	2.675620	-1.282509
H	-1.537458	2.648037	2.412014
H	-2.973733	0.249279	2.693411
C	-4.948316	0.626167	1.946305
H	-3.343343	0.580002	-1.571256
C	-5.154072	0.839028	-0.443621
H	-5.396476	0.631659	2.935624
C	-5.738045	0.847533	0.820720
H	-5.764788	1.006552	-1.326093
H	-6.803926	1.024711	0.928809
C	-0.939796	0.255294	-3.030302
C	-1.151124	-0.876680	-3.706785
H	-1.565822	1.126594	-3.199786
H	-0.135125	0.352893	-2.301882
H	-1.988992	-0.929759	-4.402249
C	-0.348562	-2.108528	-3.588385
C	-0.905239	-3.331021	-3.987729
C	0.959258	-2.099214	-3.084921
C	-0.189311	-4.517081	-3.856774
H	-1.913810	-3.347543	-4.395063
C	1.673308	-3.284416	-2.947039
H	1.427675	-1.162856	-2.794271
C	1.100419	-4.497001	-3.328534
H	-0.637573	-5.456209	-4.167846
H	2.677771	-3.250181	-2.535188
H	1.660076	-5.421732	-3.224682

=== **TSA** ===

B	1.868627	0.091660	0.254000
C	3.436175	0.130150	0.262828
C	1.009607	-1.157082	0.283901
C	0.976447	1.308423	0.377868
C	4.132649	1.316574	-0.038219
C	4.212738	-1.026988	0.449975
N	-0.326647	-1.163865	0.126208
O	1.494803	-2.406415	0.445280
N	-0.361024	1.299507	0.219483
O	1.438742	2.560354	0.608358
H	3.575778	2.235248	-0.196656
C	5.518477	1.344175	-0.159723
H	3.719823	-1.967322	0.671823
C	5.600488	-1.004731	0.336218
B	-1.040618	0.072143	-0.270165
C	-0.845035	-2.547166	0.325050
C	0.457707	-3.340585	0.148719
C	-0.875480	2.700463	0.240185
C	0.325112	3.395577	0.896964
H	6.021311	2.276913	-0.401321
C	6.261564	0.179915	0.024780
H	6.166430	-1.920827	0.483861
C	-2.634740	0.069411	-0.318639
C	-1.361017	-2.707187	1.757808
C	-1.896367	-2.988471	-0.686684
H	0.538263	-4.178649	0.844788
H	0.590228	-3.695571	-0.878536
C	-1.101406	3.222959	-1.178697
C	-2.123515	2.913837	1.091474
H	0.516518	4.392517	0.494022
H	0.202943	3.454615	1.986222
H	7.343571	0.197801	-0.071296
C	-3.346313	-0.006734	0.891032
C	-3.392575	0.142849	-1.493986
H	-2.018642	-4.075154	-0.613696
H	-2.865905	-2.524446	-0.496922
H	-0.592119	-2.402716	2.475153
H	-1.613961	-3.756917	1.942651
H	-2.258986	-2.108717	1.919890
H	-1.585966	-2.748391	-1.707599
H	-2.228901	3.987435	1.287690

H	-3.028643	2.571942	0.587427
H	-0.189078	3.133866	-1.778026
H	-1.388228	4.279159	-1.136962
H	-1.910752	2.673014	-1.667920
H	-2.034541	2.396931	2.051209
H	-2.793136	-0.025951	1.827872
C	-4.737051	-0.028540	0.930221
H	-2.893520	0.206979	-2.456873
C	-4.787491	0.128566	-1.468234
H	-5.253131	-0.089226	1.884210
C	-5.464587	0.036806	-0.256718
H	-5.344513	0.186278	-2.398964
H	-6.550155	0.021245	-0.235142
C	-0.307280	0.134730	-2.327130
C	1.073772	0.089308	-2.313581
H	-0.789603	1.083561	-2.527594
H	-0.865623	-0.732579	-2.669078
H	1.629820	1.024212	-2.283108
C	1.880757	-1.093334	-2.656959
C	3.280729	-0.995664	-2.702770
C	1.302995	-2.327076	-2.994099
C	4.068070	-2.090894	-3.036307
H	3.755569	-0.050974	-2.452440
C	2.090061	-3.423466	-3.328540
H	0.221844	-2.430099	-3.020149
C	3.478876	-3.314309	-3.345084
H	5.148693	-1.984642	-3.043920
H	1.614288	-4.365216	-3.589251
H	4.092598	-4.170613	-3.607044

=== **TSB** ===

B	1.687401	-0.177755	-0.185519
C	3.260722	-0.189461	-0.289554
C	0.819026	-1.397884	0.133840
C	0.876205	1.072044	0.123000
C	3.942544	0.915014	-0.828703
C	4.041158	-1.291850	0.095325
N	-0.516284	-1.355734	0.154031
O	1.288740	-2.638993	0.368134
N	-0.467431	1.106081	0.147340
O	1.411813	2.304550	0.225163
H	3.376317	1.790315	-1.136359
C	5.326121	0.923124	-0.975750
H	3.556730	-2.166937	0.516613
C	5.426688	-1.290350	-0.046650
B	-1.238575	-0.111601	-0.178950
C	-1.056709	-2.687630	0.568486
C	0.189525	-3.555024	0.336006
C	-0.925628	2.526621	0.114071
C	0.369589	3.213698	0.568929
H	5.820115	1.793906	-1.398282
C	6.076326	-0.182812	-0.583896
H	6.001216	-2.159420	0.262557
C	-2.823277	-0.057819	-0.088686
C	-1.389610	-2.653603	2.062413
C	-2.243104	-3.185169	-0.250743
H	0.339940	-4.299428	1.120525
H	0.167585	-4.039762	-0.644710
C	-1.302285	2.929561	-1.311785
C	-2.055865	2.860289	1.082209
H	0.552575	4.165372	0.065556
H	0.381502	3.361998	1.655865
H	7.156676	-0.181172	-0.697333
C	-3.450119	-0.008765	1.168277
C	-3.656087	-0.050518	-1.212404
H	-2.386578	-4.250204	-0.033982
H	-3.162800	-2.660593	0.017274
H	-0.520604	-2.328372	2.643747
H	-1.675194	-3.657577	2.394343
H	-2.225954	-1.982170	2.261362
H	-2.066124	-3.072467	-1.323420
H	-2.101353	3.949060	1.201971
H	-3.023748	2.519797	0.712263
H	-0.470472	2.761514	-2.003233
H	-1.566448	3.992239	-1.336636
H	-2.168829	2.354373	-1.650969
H	-1.873331	2.414393	2.064190
H	-2.837664	0.033958	2.066547
C	-4.835246	0.018883	1.298712
H	-3.209589	-0.083581	-2.199263
C	-5.045166	-0.017820	-1.096628
H	-5.287105	0.056731	2.285836
C	-5.639873	0.009107	0.160520
H	-5.661900	-0.017360	-1.990644
H	-6.721437	0.029777	0.255467
C	0.926553	0.161399	-2.258387
C	-0.408426	-0.191257	-2.476653
H	1.208855	1.211220	-2.286522
H	1.723200	-0.494420	-2.597017
H	-1.117462	0.595031	-2.724219
C	-0.726030	-1.527925	-2.998420
C	-1.897609	-1.766026	-3.738762
C	0.144963	-2.616877	-2.813945
C	-2.207386	-3.028321	-4.229373
H	-2.563784	-0.935318	-3.957261
C	-0.160142	-3.879623	-3.314810
H	1.076607	-2.476498	-2.270435
C	-1.343646	-4.101431	-4.013304
H	-3.123889	-3.172884	-4.794482
H	0.539133	-4.697113	-3.156898
H	-1.581233	-5.087699	-4.399028

=== **4a(A)** ===

B	1.787110	0.047439	-0.211651
C	3.373901	0.085552	-0.021271
C	0.932665	-1.221425	0.267991
C	0.941645	1.293468	0.346571
C	4.073575	1.288396	-0.212536
C	4.147741	-1.064646	0.192529
N	-0.369412	-1.206571	0.156183
O	1.424314	-2.401615	0.638857
N	-0.362654	1.309822	0.260540
O	1.469762	2.444718	0.756358
H	3.520493	2.204439	-0.402985
C	5.464281	1.347694	-0.178232
H	3.657086	-2.025330	0.311116
C	5.539790	-1.015882	0.230727
B	-0.984926	0.082275	-0.565041
C	-0.930814	-2.530156	0.545662
C	0.363267	-3.367591	0.544501
C	-0.855917	2.702452	0.445505
C	0.391959	3.331950	1.091247
H	5.968792	2.298079	-0.331416
C	6.206366	0.191670	0.048221
H	6.104870	-1.929286	0.396449
C	-2.592290	0.076794	-0.586411
C	-1.516965	-2.478926	1.958088
C	-1.950731	-3.071061	-0.452632
H	0.438984	-4.046115	1.395864
H	0.506212	-3.921543	-0.390044
C	-1.154598	3.313006	-0.924758
C	-2.053544	2.850125	1.377180
H	0.627758	4.325308	0.704100
H	0.314345	3.368083	2.183347

H	7.291676	0.231471	0.075960
C	-3.361503	0.067812	0.587811
C	-3.295378	0.023345	-1.797290
H	-2.116158	-4.136012	-0.253870
H	-2.908529	-2.553332	-0.365655
H	-0.781532	-2.083910	2.666291
H	-1.794773	-3.490655	2.273295
H	-2.413215	-1.859220	1.987558
H	-1.591433	-2.963130	-1.480586
H	-2.161756	3.908120	1.643079
H	-2.978892	2.523825	0.901151
H	-0.262483	3.296479	-1.559982
H	-1.482745	4.351419	-0.807040
H	-1.950633	2.751722	-1.423414
H	-1.903314	2.279290	2.298381
H	-2.855878	0.140646	1.548888
C	-4.751477	-0.012855	0.564700
H	-2.742455	0.032543	-2.733388
C	-4.687646	-0.053383	-1.839092
H	-5.313503	-0.020435	1.494634
C	-5.420346	-0.079196	-0.656534
H	-5.199553	-0.097473	-2.796336
H	-6.504085	-0.144931	-0.683895
C	-0.225774	0.124949	-2.019652
C	1.310617	0.081563	-1.870365
H	-0.521416	1.030813	-2.564676
H	-0.573136	-0.710339	-2.640231
H	1.754804	1.012097	-2.245434
C	2.010231	-1.063190	-2.560349
C	3.310265	-0.897642	-3.055560
C	1.442121	-2.338661	-2.658203
C	4.008301	-1.955951	-3.626224
H	3.785678	0.076450	-2.967750
C	2.136496	-3.403879	-3.226435
H	0.431435	-2.503679	-2.292505
C	3.425700	-3.218199	-3.715112
H	5.015361	-1.793930	-3.999538
H	1.663024	-4.380060	-3.294333
H	3.968366	-4.044993	-4.163230

=== **4a(B)** ===

B	1.595422	-0.143617	-0.641806
C	3.187444	-0.137958	-0.509937
C	0.827363	-1.437757	-0.082653
C	0.812938	1.067163	0.054042
C	4.005947	0.346447	-1.537227
C	3.827194	-0.592390	0.652305
N	-0.474514	-1.449659	0.027418
O	1.409915	-2.601381	0.191059
N	-0.494533	1.075031	0.049977
O	1.364946	2.221487	0.405887
H	3.549527	0.707628	-2.456459
C	5.394095	0.378973	-1.416496
H	3.226208	-0.982395	1.472329
C	5.212110	-0.567199	0.786500
B	-1.187375	-0.186137	-0.657359
C	-0.921645	-2.790428	0.526457
C	0.369894	-3.593903	0.282821
C	-0.971582	2.479148	0.205017
C	0.301310	3.105255	0.801001
H	6.001368	0.759544	-2.233395
C	6.002442	-0.079700	-0.252027
H	5.676439	-0.930126	1.699583
C	-2.772142	-0.091961	-0.398449
C	-1.206092	-2.749729	2.033481
C	-2.104795	-3.377371	-0.234807
H	0.618070	-4.272549	1.100293
H	0.343936	-4.137099	-0.667093
C	-1.277930	3.056286	-1.178534
C	-2.154891	2.675671	1.146066
H	0.518533	4.102559	0.414009
H	0.271489	3.127807	1.896165
H	7.084120	-0.059242	-0.153679
C	-3.270798	-0.251253	0.903913
C	-3.699656	0.286008	-1.375280
H	-2.284097	-4.394853	0.131543
H	-3.009818	-2.790563	-0.062924
H	-0.396556	-2.243542	2.569394
H	-1.278975	-3.776057	2.408787
H	-2.150241	-2.253720	2.256192
H	-1.910771	-3.424449	-1.308745
H	-2.229030	3.740990	1.394957
H	-3.095199	2.367729	0.684716
H	-0.404561	2.981981	-1.835503
H	-1.558937	4.111280	-1.088445
H	-2.111313	2.516907	-1.637519
H	-2.016075	2.112897	2.073399
H	-2.566395	-0.445998	1.707964
C	-4.618631	-0.100956	1.212286
H	-3.358123	0.480358	-2.387870
C	-5.054162	0.457227	-1.083904
H	-4.963632	-0.234099	2.234038
C	-5.521598	0.249040	0.209414
H	-5.742768	0.754696	-1.869777
H	-6.575769	0.374229	0.438524
C	0.930762	0.095629	-2.172530
C	-0.582527	-0.221984	-2.229877
H	1.102105	1.148132	-2.434630
H	1.493794	-0.482846	-2.912244
H	-1.101976	0.553529	-2.810150
C	-0.857854	-1.550693	-2.908862
C	-2.076975	-1.798034	-3.555407
C	0.073998	-2.600345	-2.900628
C	-2.371022	-3.032471	-4.126189
H	-2.815443	-1.006427	-3.621698
C	-0.211420	-3.838161	-3.474241
H	1.050227	-2.458440	-2.443991
C	-1.441649	-4.068412	-4.081916
H	-3.330425	-3.181138	-4.613507
H	0.539681	-4.623697	-3.447843
H	-1.665541	-5.032060	-4.528981

5. References

[1] a) Hammett, L. P. *J. Am. Chem. Soc.* **1937**, *59*, 96 – 103; b) Hansch, C.; Leo, A.; Taft, R. W. *Chem. Rev.* **1991**, *91*, 165 – 195; c) Brown, T. J.; Dichens, M. G.; Widenhoefer, R. A. *J. Am. Chem. Soc.* **2009**, *131*, 6350 – 6351.

[2] G. M. Sheldrick, SHELXL-97, Program for Crystal Structure Refinement; University of Göttingen: Göttingen, Germany, **1997**.

[3] Bruker AXS SHELXTL, Madison, WI; SHELX-97G. M. Sheldrick, *Acta Crystallogr. A*, **2008**, *64*, 112–122, SHELX-2013, http://shelx.uni-ac.gwdg.de/SHELX/index.php.

[4] Complete List of Authors of Gaussian 09 **(**Gaussian 09, Revision B.01); M. J. Frisch, G. W. Trucks, H. B. Schlegel, G. E. Scuseria, M. A. Robb, J. R. Cheeseman, G. Scalmani, V. Barone, B. Mennucci, G. A. Petersson, H. Nakatsuji, M. Caricato, X. Li, H. P. Hratchian, A. F. Izmaylov, J. Bloino, G. Zheng, J. L. Sonnenberg, M. Hada, M. Ehara, K. Toyota, R. Fukuda, J. Hasegawa, M. Ishida, T. Nakajima, Y. Honda, O. Kitao, H. Nakai, T. Vreven, J. A. Montgomery, Jr., J. E. Peralta, F. Ogliaro, M. Bearpark, J. J. Heyd, E. Brothers, K. N. Kudin, V. N. Staroverov, T. Keith, R. Kobayashi, J. Normand, K. Raghavachari, A. Rendell, J. C. Burant, S. S. Iyengar, J. Tomasi, M. Cossi, N. Rega, J. M. Millam, M. Klene, J. E. Knox, J. B. Cross, V. Bakken, C. Adamo, J. Jaramillo, R. Gomperts, R. E. Stratmann, O. Yazyev, A. J. Austin, R. Cammi, C. Pomelli, J. W. Ochterski, R. L. Martin, K. Morokuma, V. G. Zakrzewski, G. A. Voth, P. Salvador, J. J. Dannenberg, S. Dapprich, A. D. Daniels, O. Farkas, J. B. Foresman, J. V. Ortiz, J. Cioslowski, and D. J. Fox, Gaussian, Inc., Wallingford CT, 2010.

[5] Zhao, Y.; Truhlar, D. G. *Theor. Chem. Acc.* **2008**, *120*, 215 – 241.

[6] a) Hehre, W. R.; Schleyer, P. v. R.; Pople, J. A. *Ab Initio Molecular Orbital Theory*, John Wiley & Sons, New York, **1986**.

[7] Tomasi, J.; Mennucci, B.; Cammi, R. *Chem. Rev*. **2005**, *105*, 2999.

[8] Weigend, F.; Ahlrichs, R. *Phys. Chem. Chem. Phys.* **2005**, *7*, 3297.

5. NMR spectra

Compound 2

^{1}H NMR

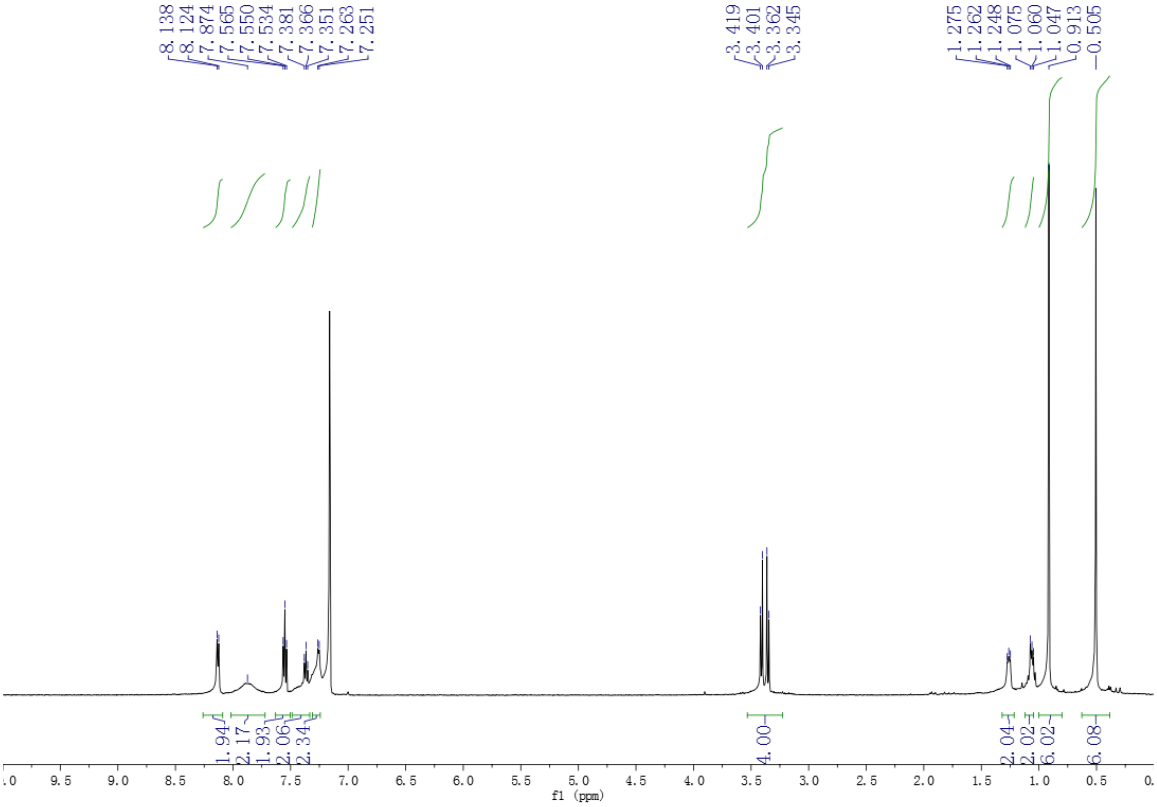

^{13}C NMR

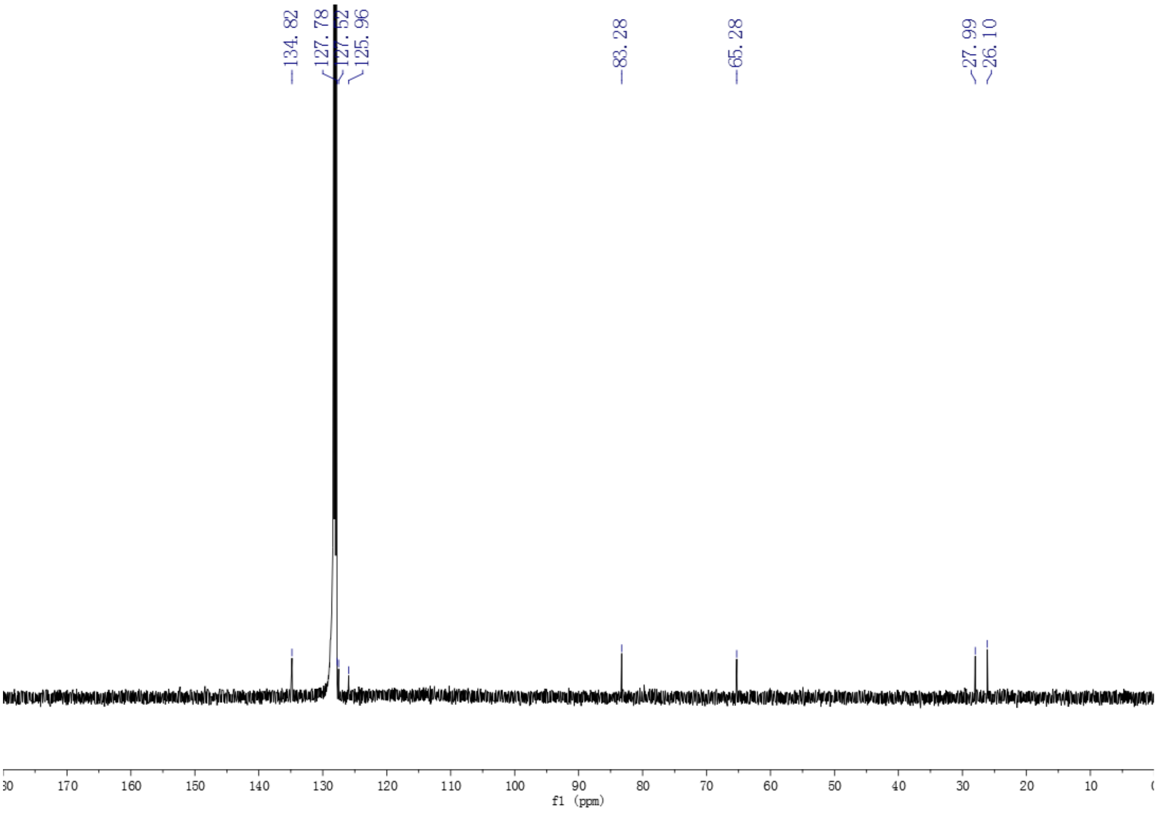

^{13}C NMR (DEPT 135)

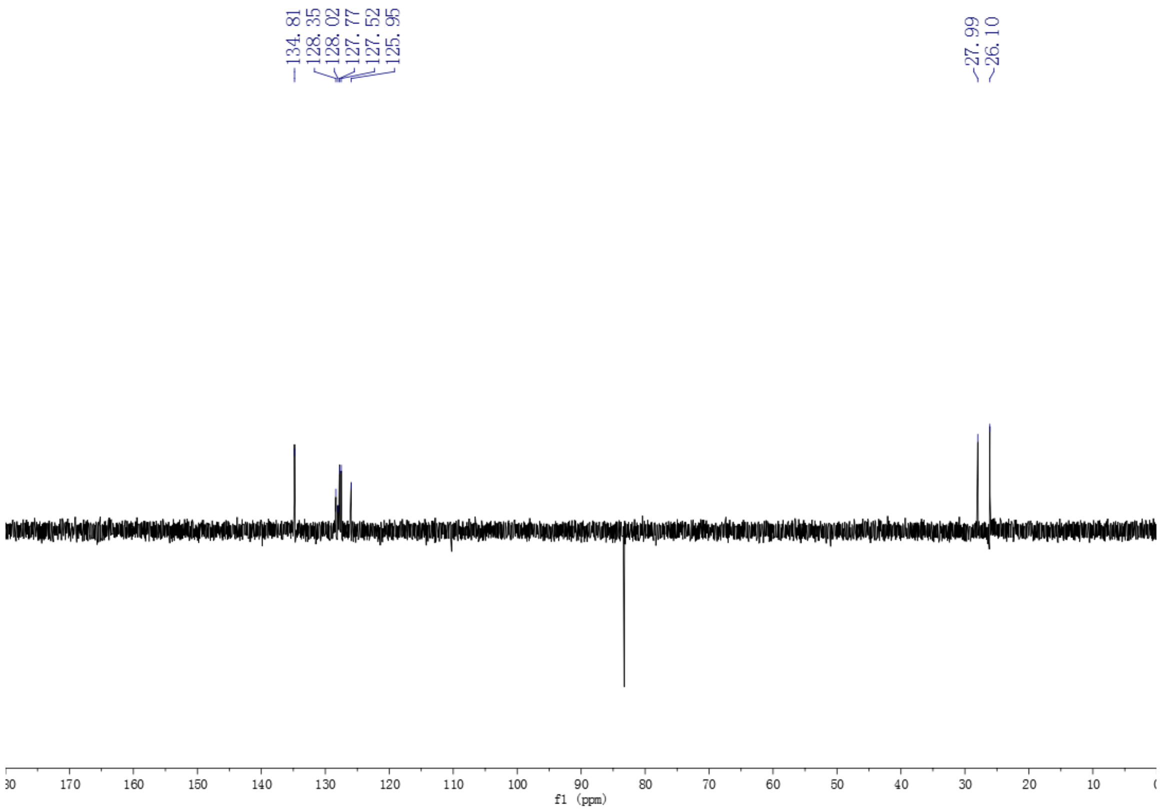

^{11}B NMR

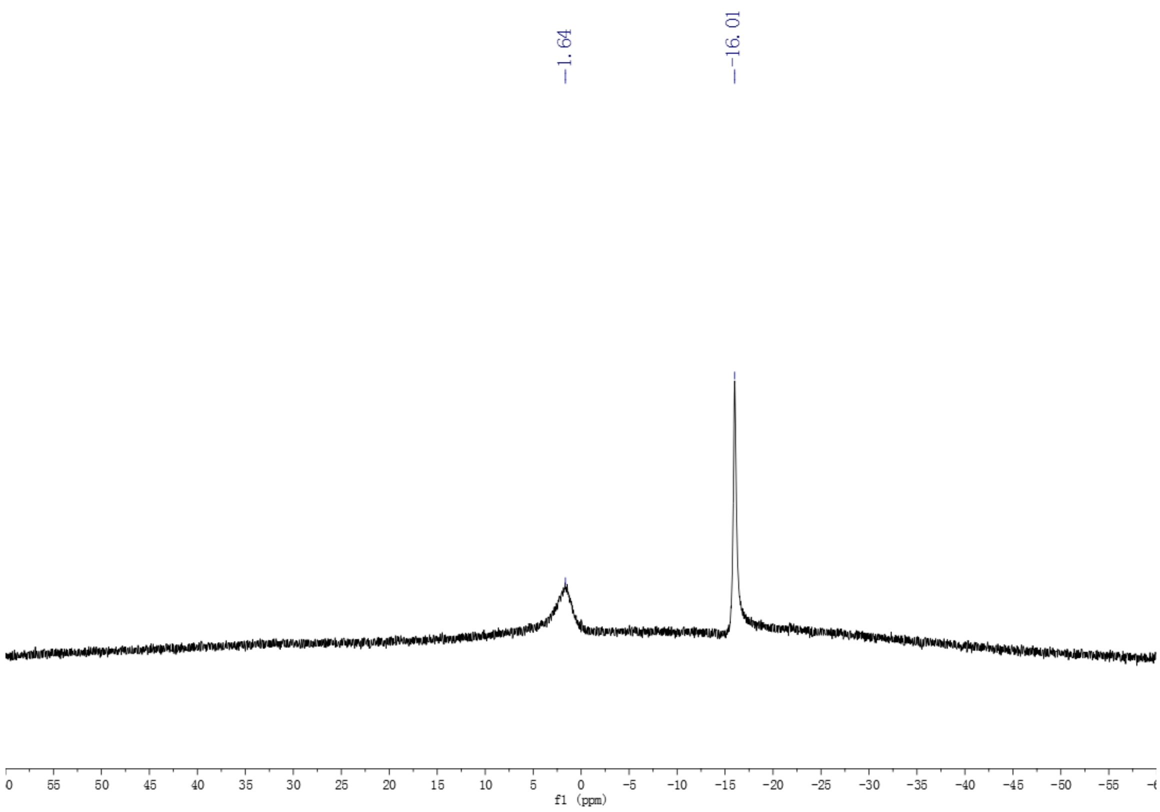

Compound 4a

^{1}H NMR

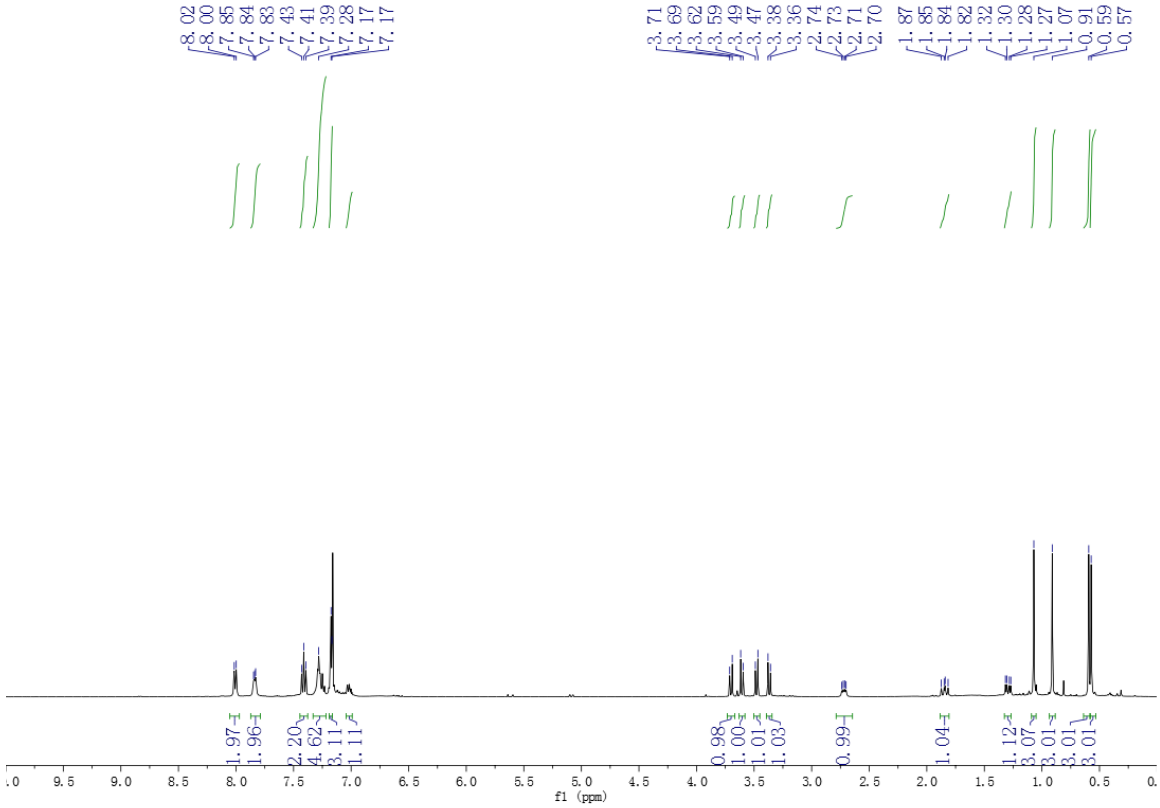

^{13}C NMR

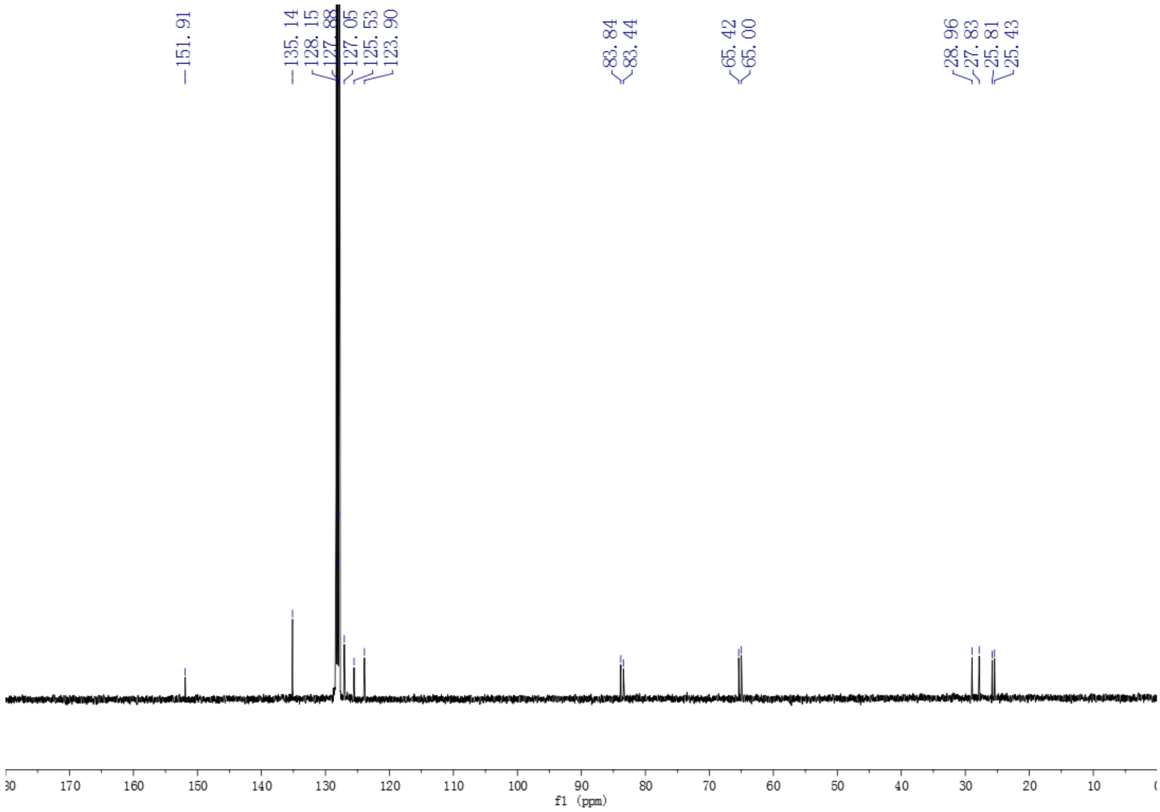

^{13}C NMR (DEPT 135)

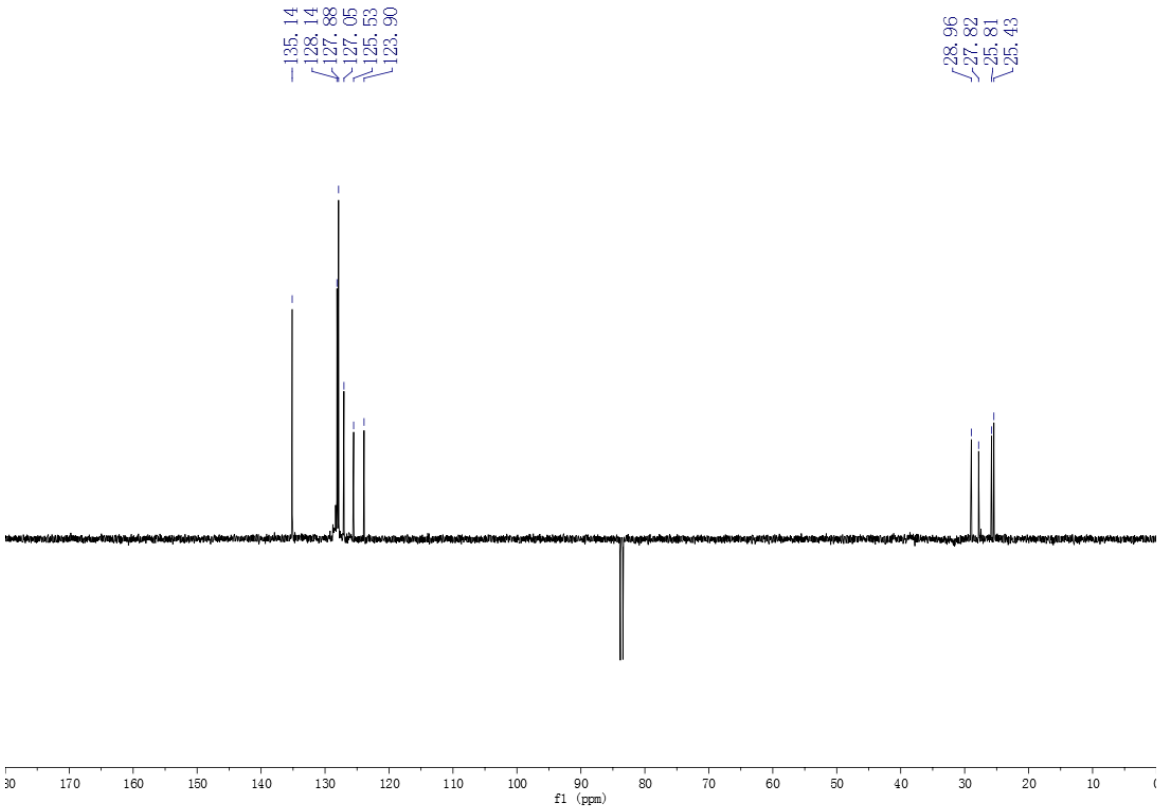

^{11}B NMR

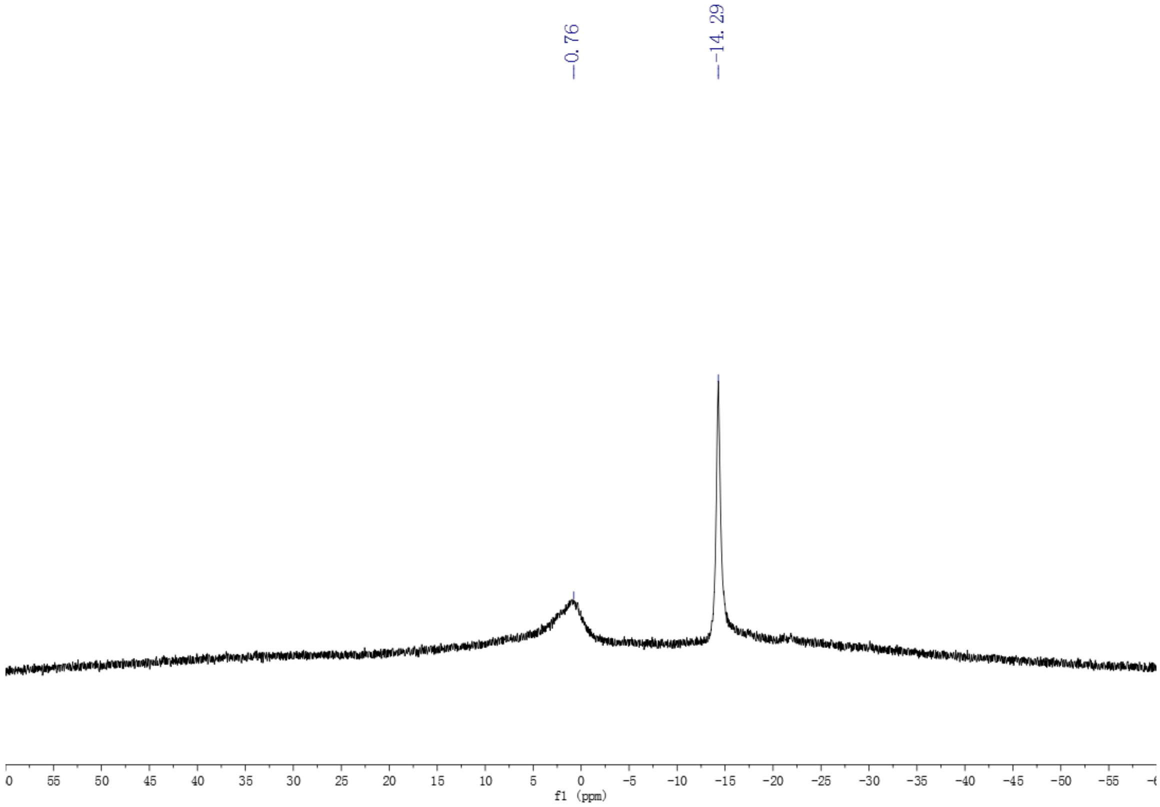

Compound 4b

^{1}H NMR

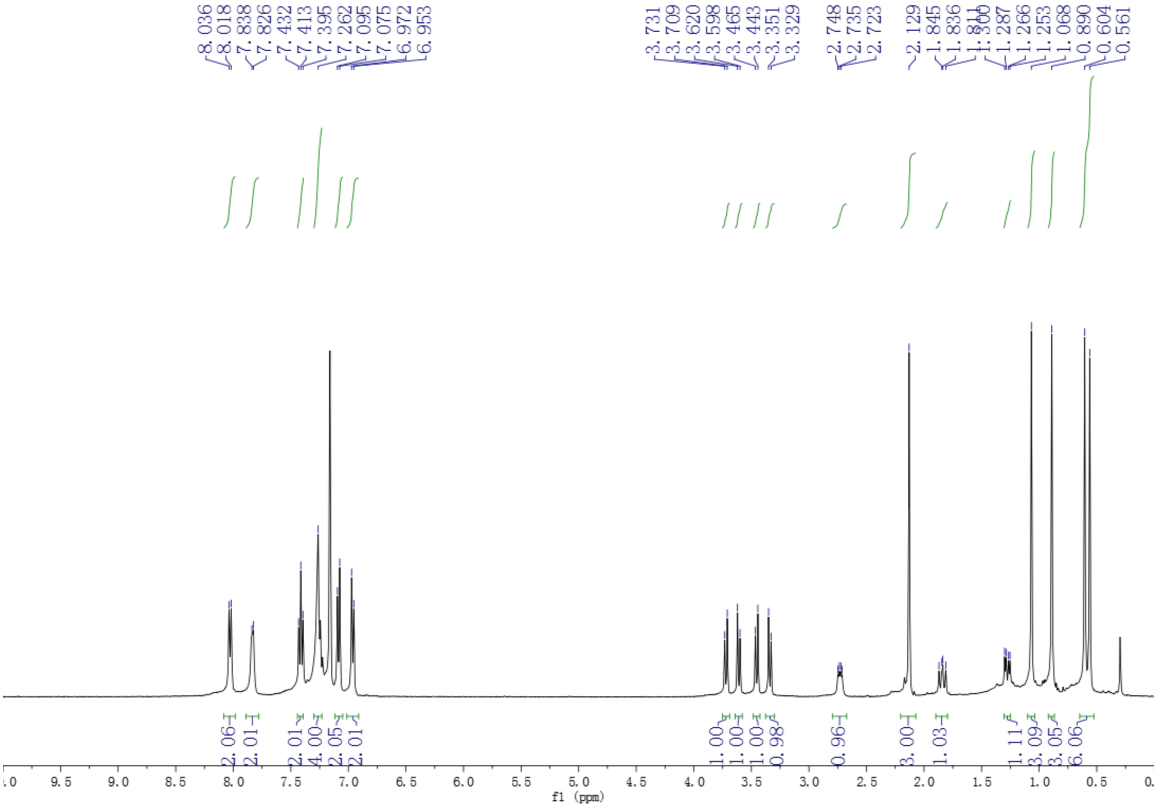

^{13}C NMR

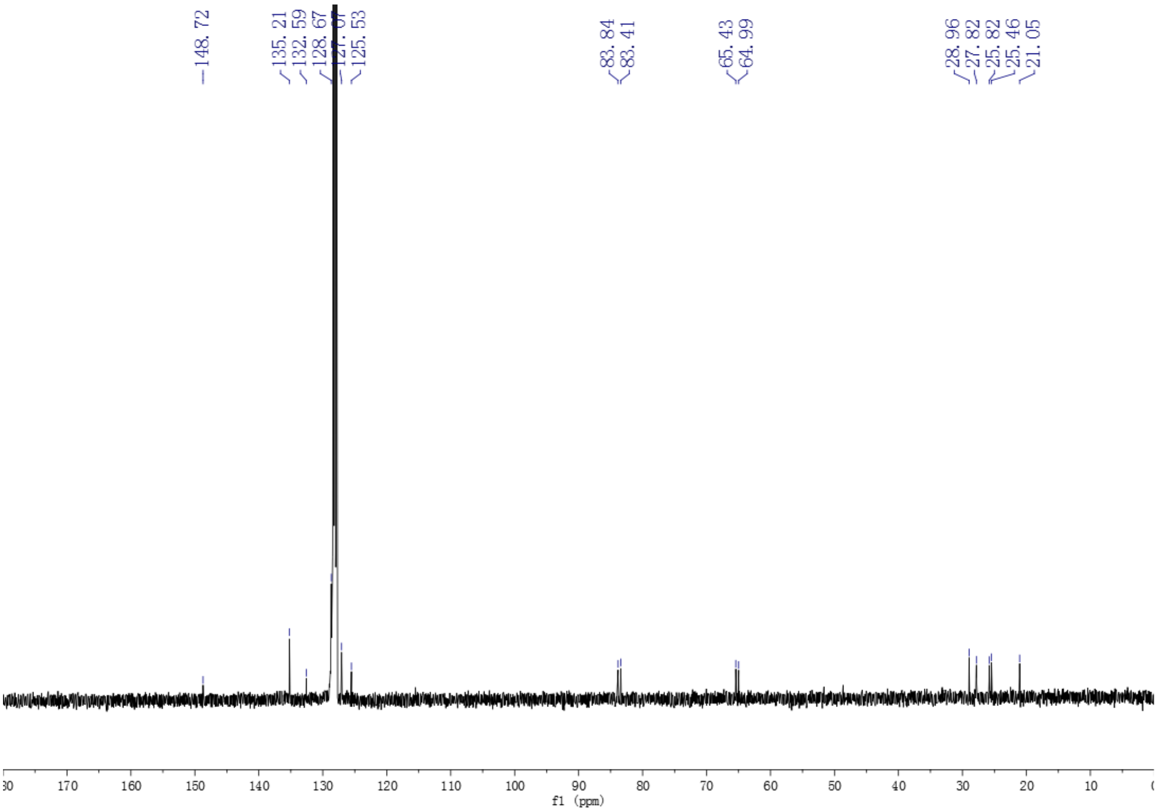

^{13}C NMR (DEPT 135)

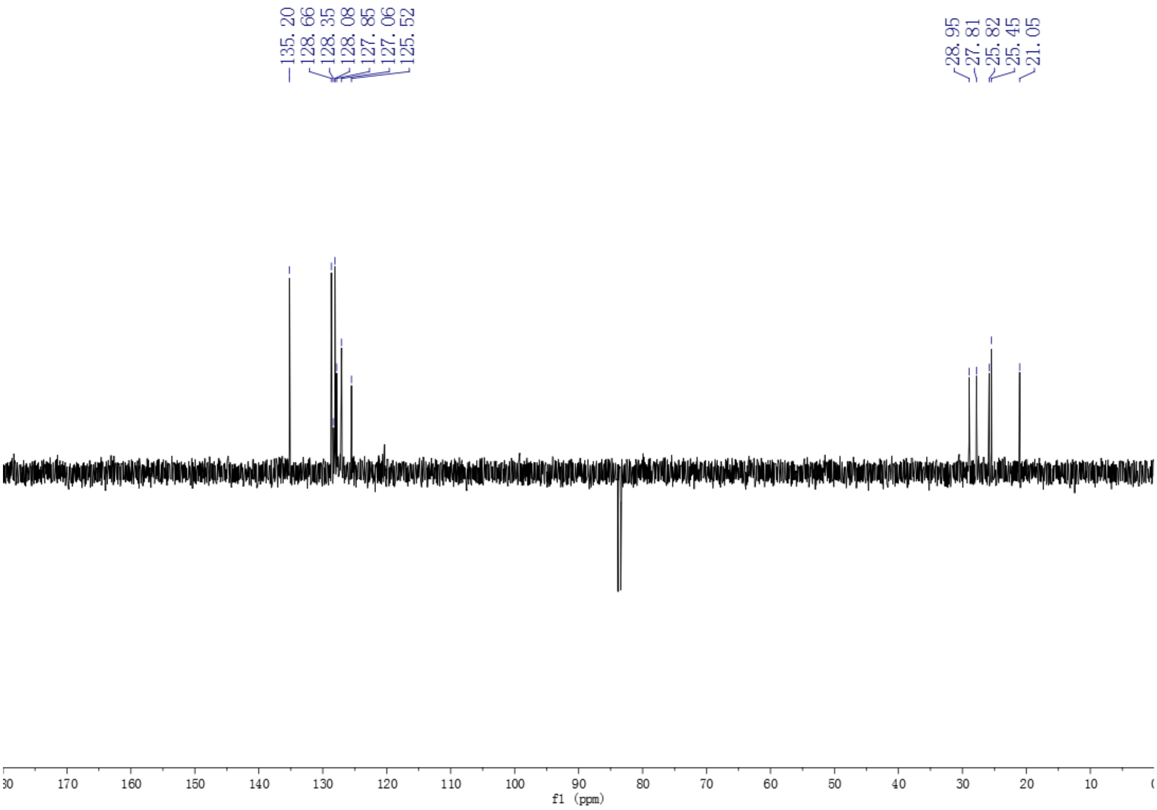

^{11}B NMR

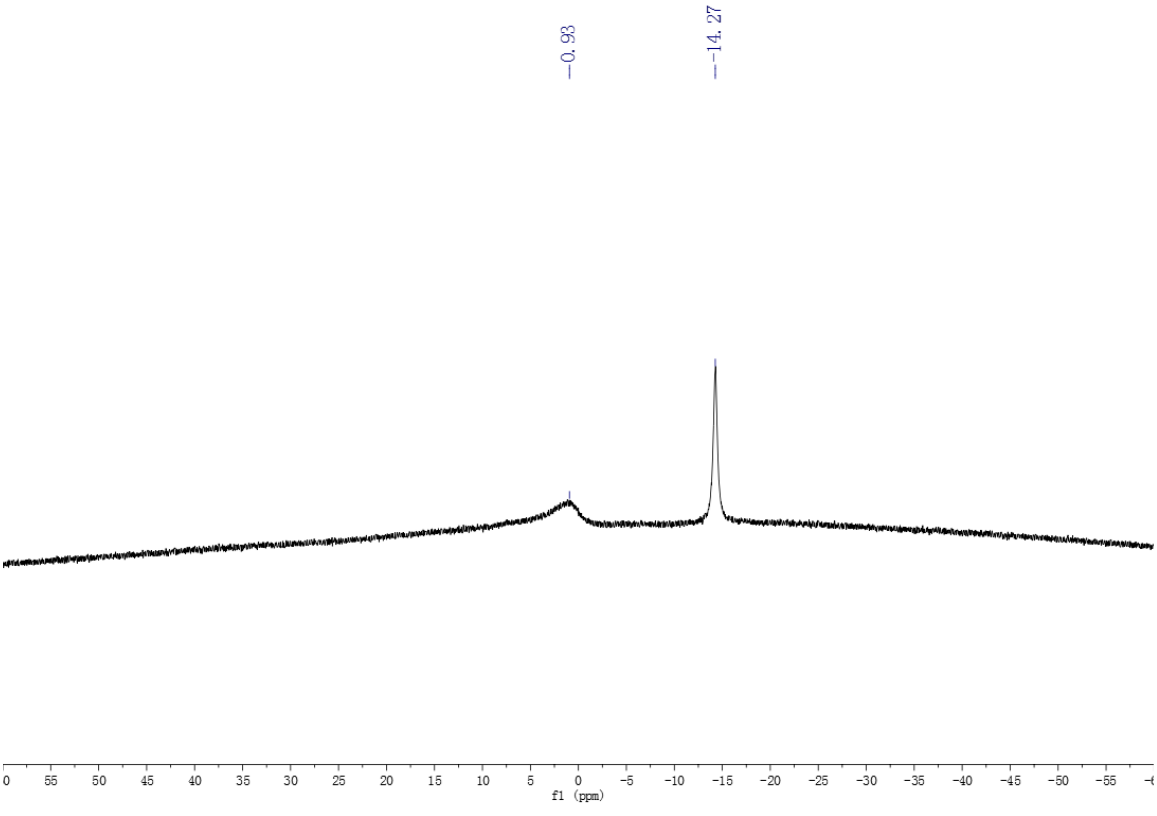

Compound 4c

^{1}H NMR

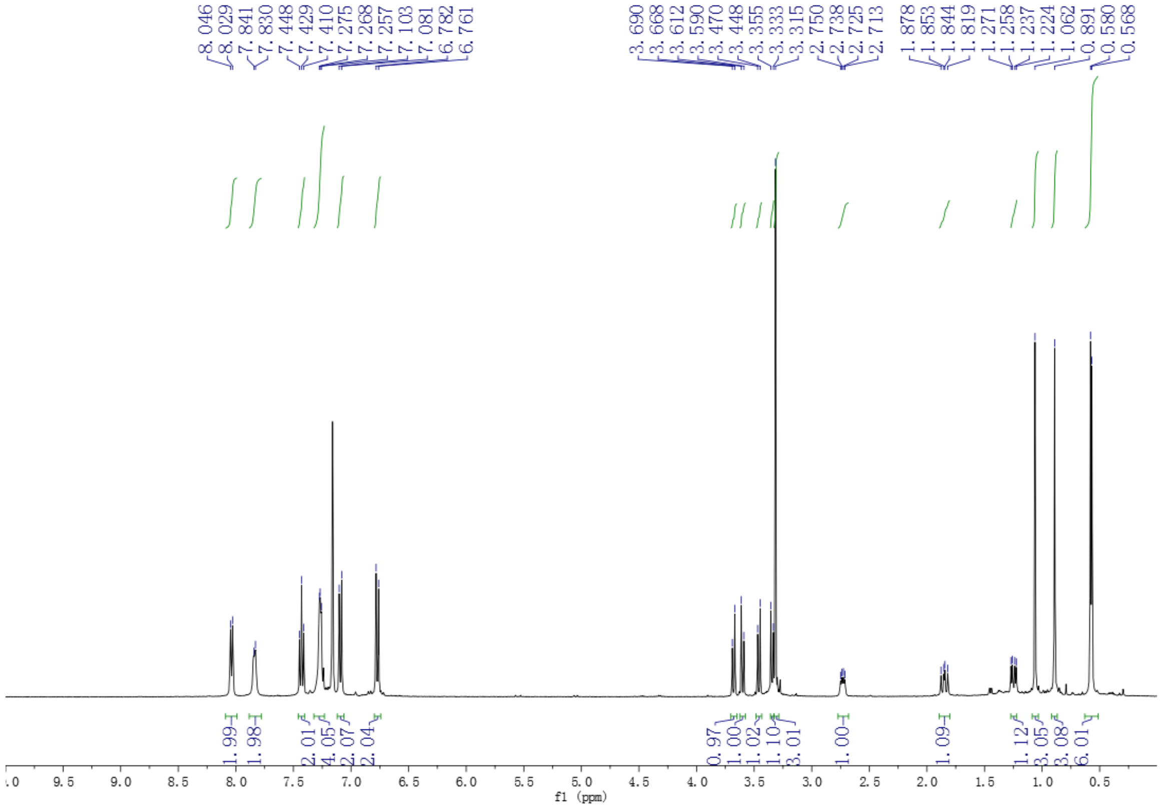

^{13}C NMR

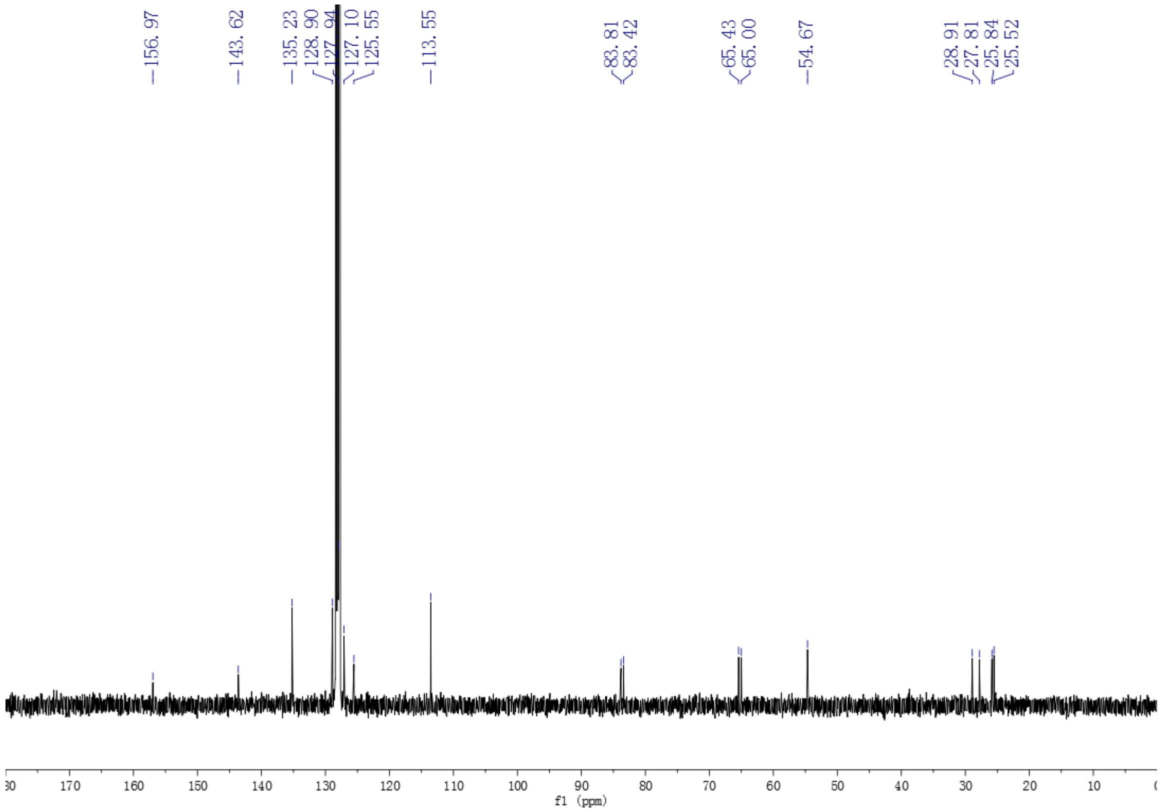

^{13}C NMR (DEPT 135)

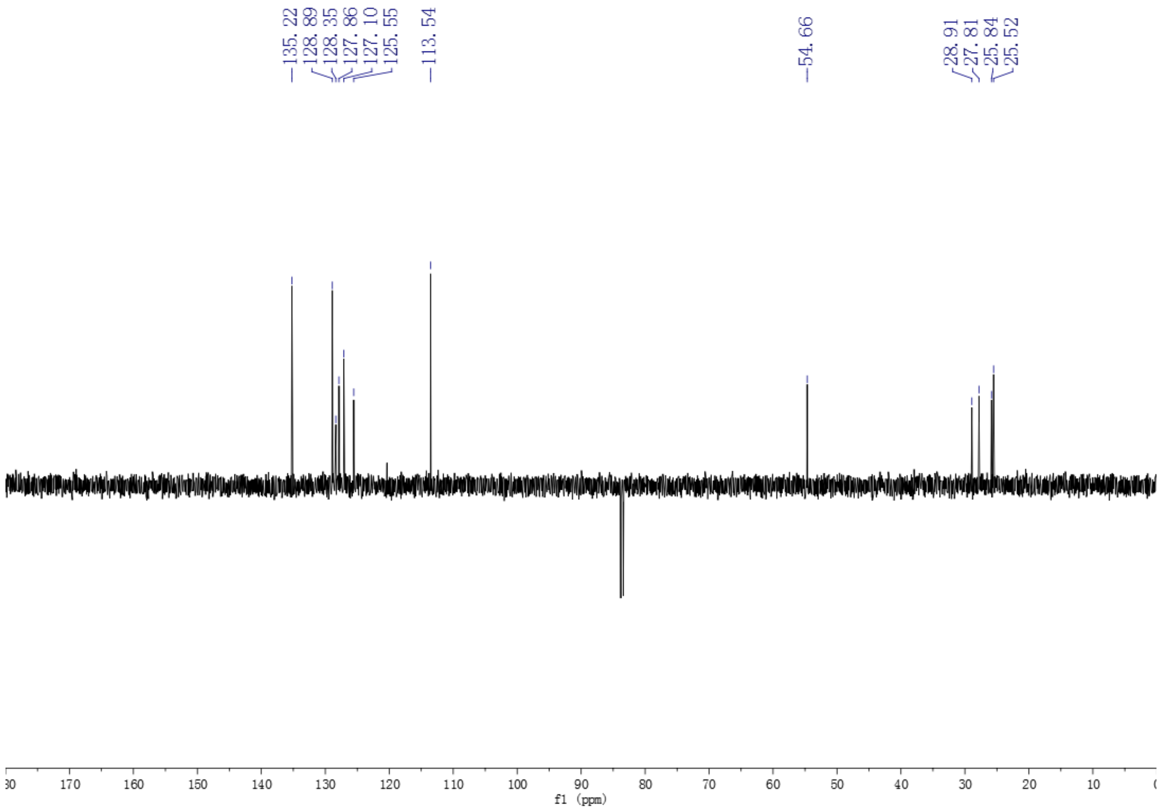

^{11}B NMR

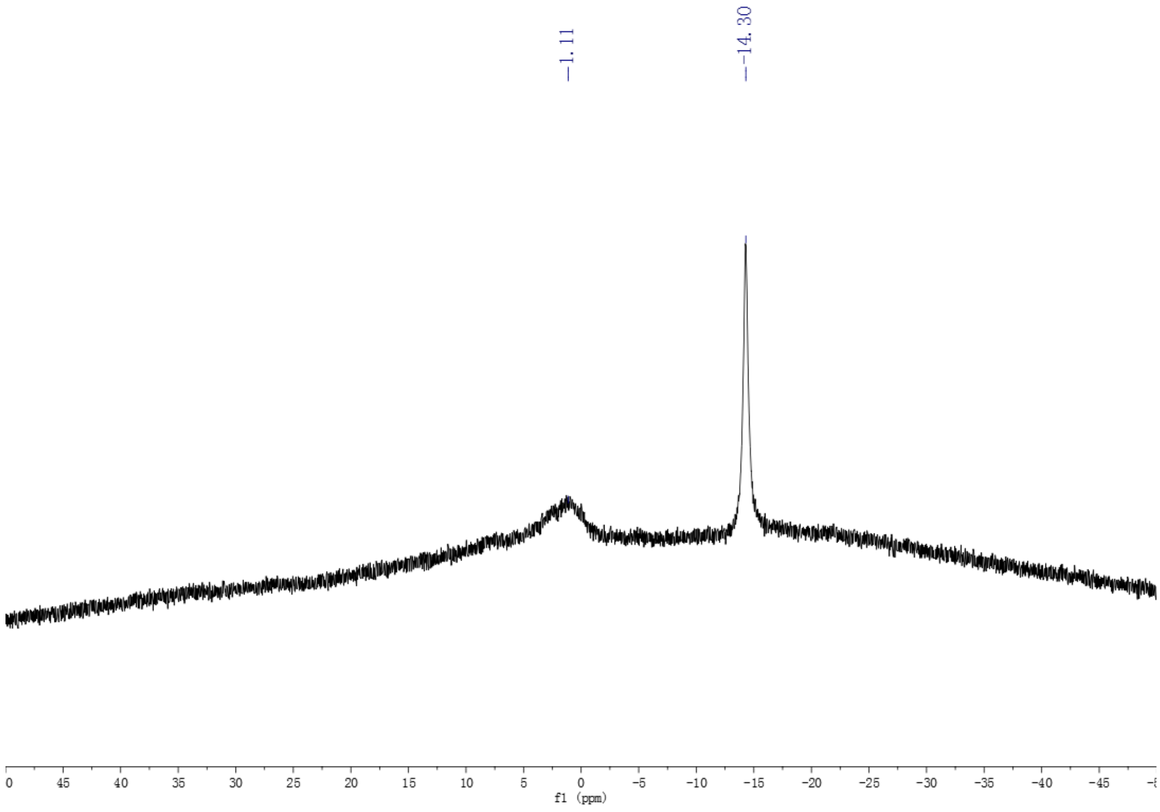

Compound 4d

^{1}H NMR

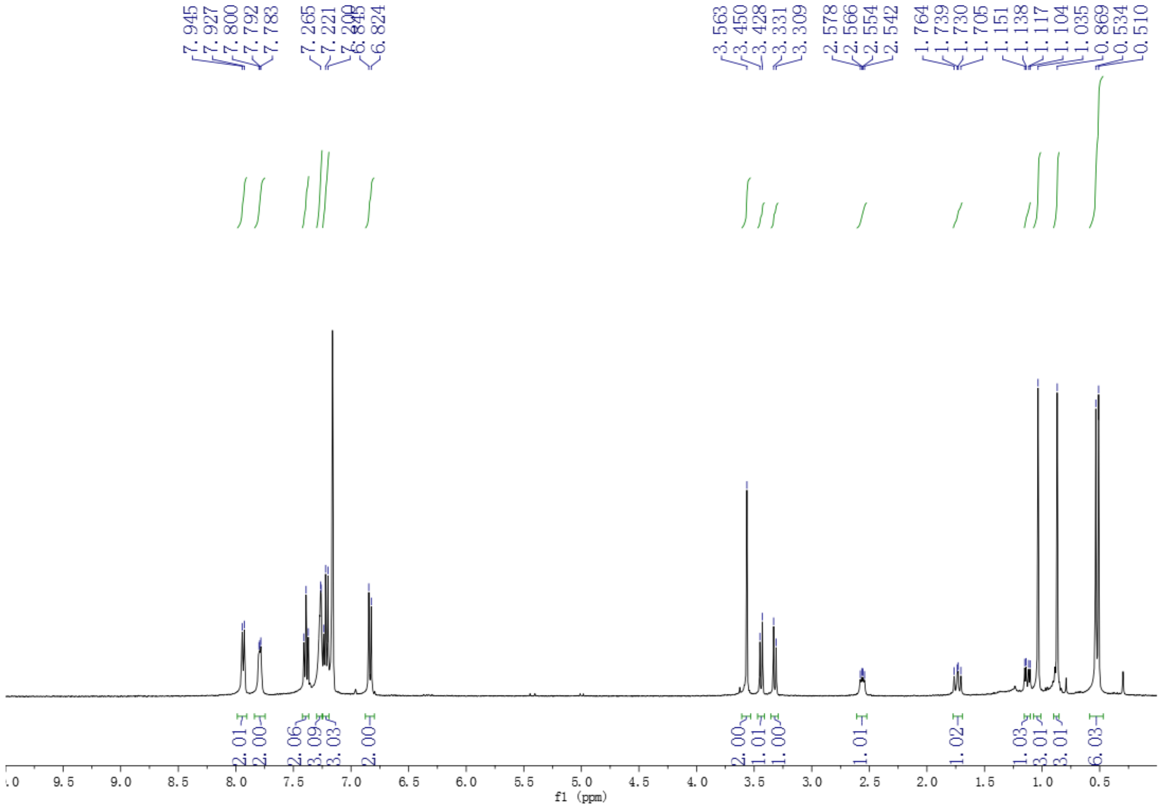

^{13}C NMR

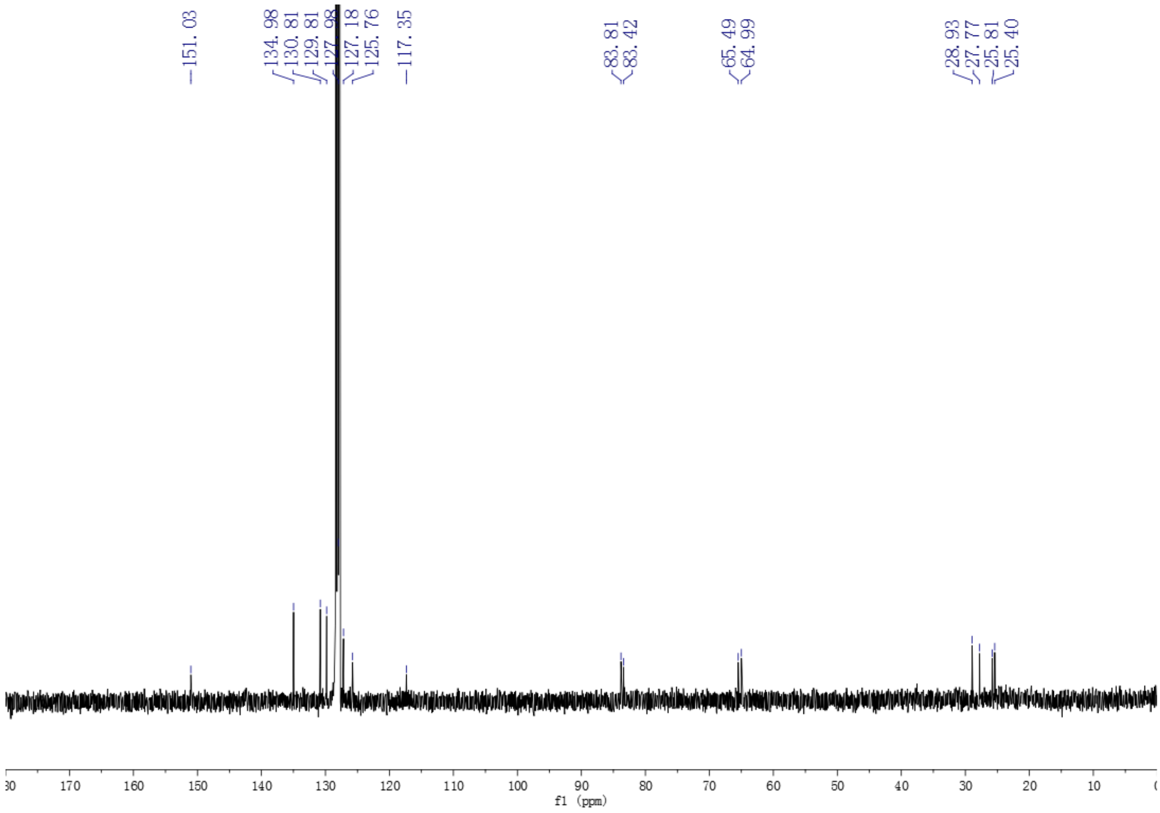

^{13}C NMR (DEPT 135)

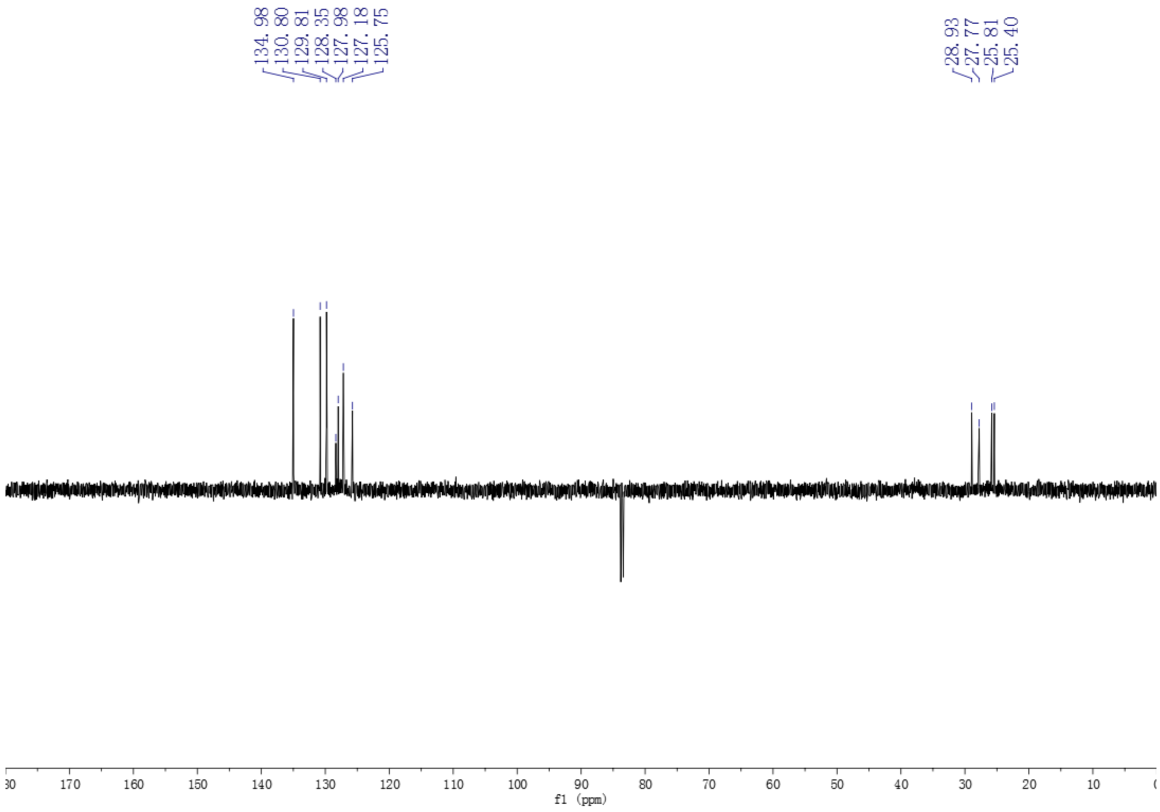

^{11}B NMR

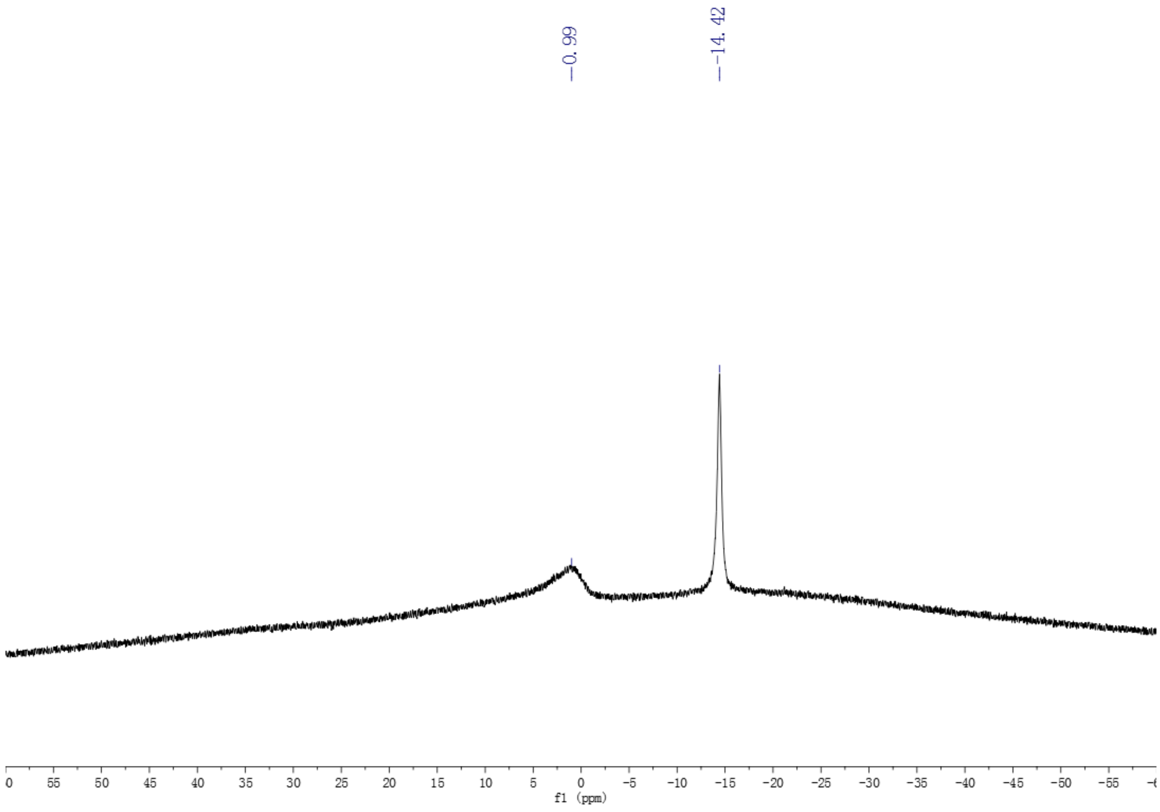

Compound 4e

^{1}H NMR

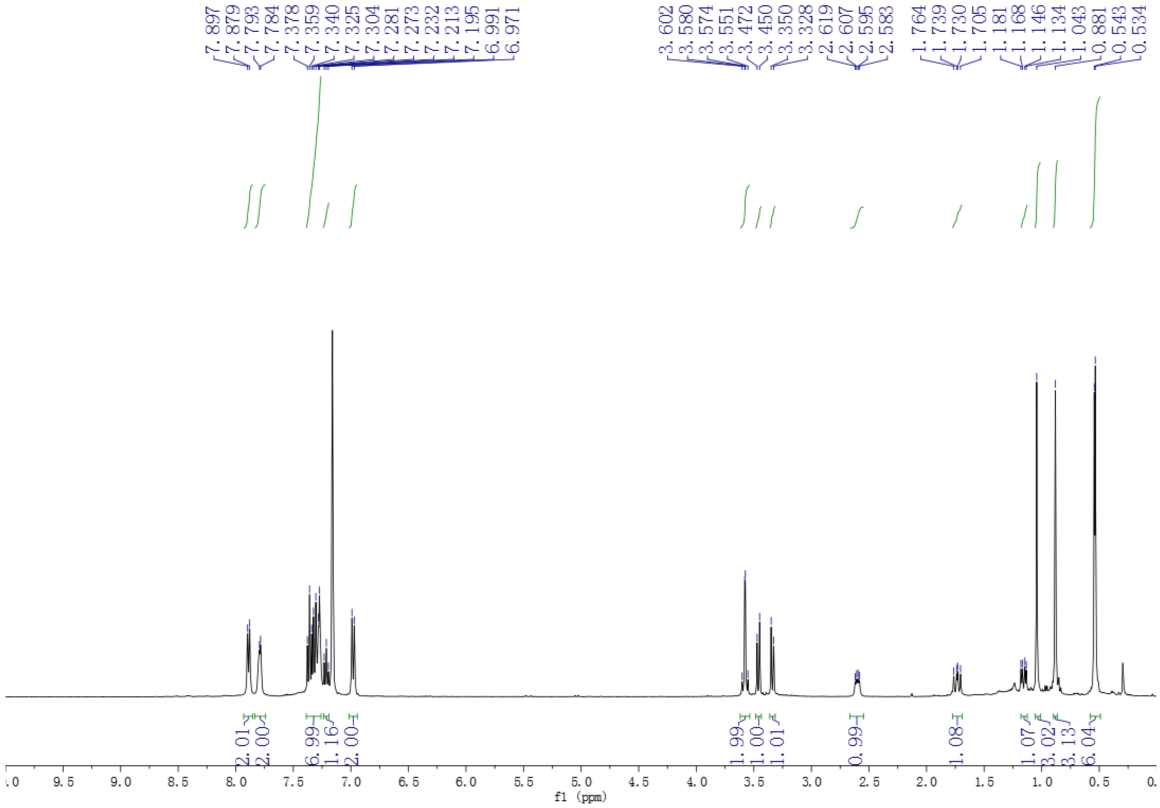

^{13}C NMR

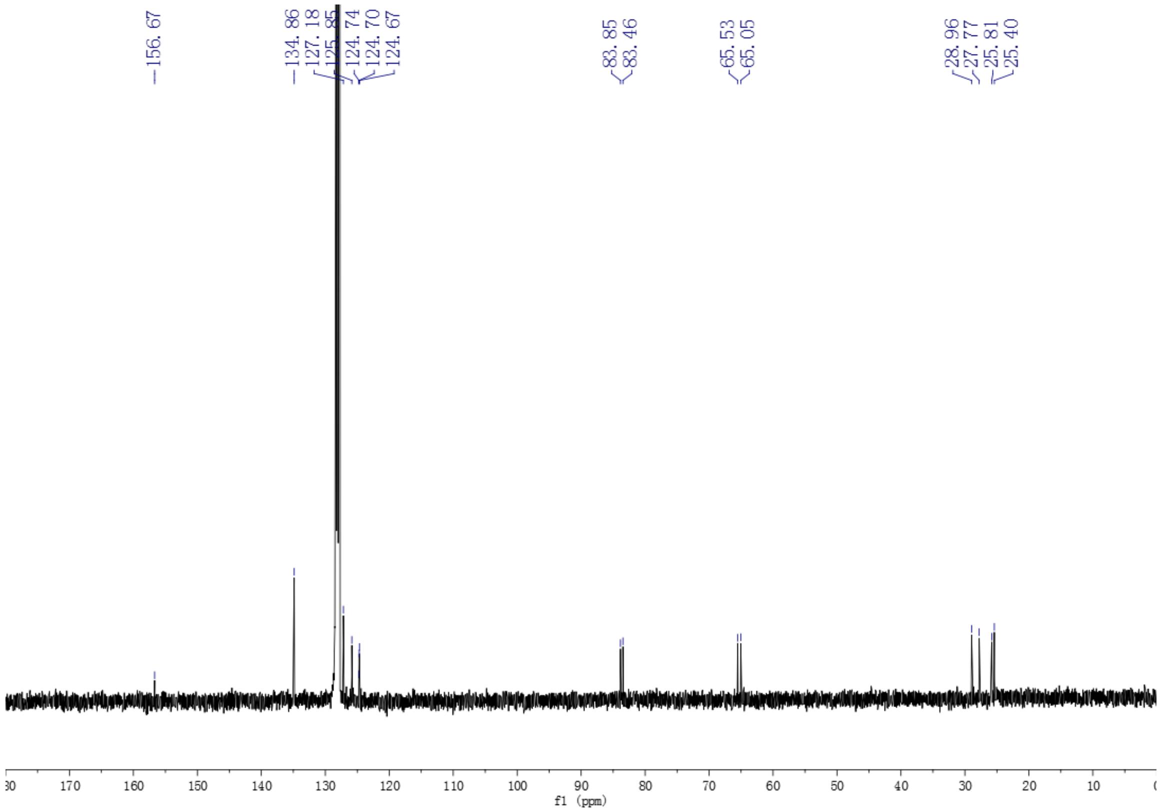

^{13}C NMR (DEPT 135)

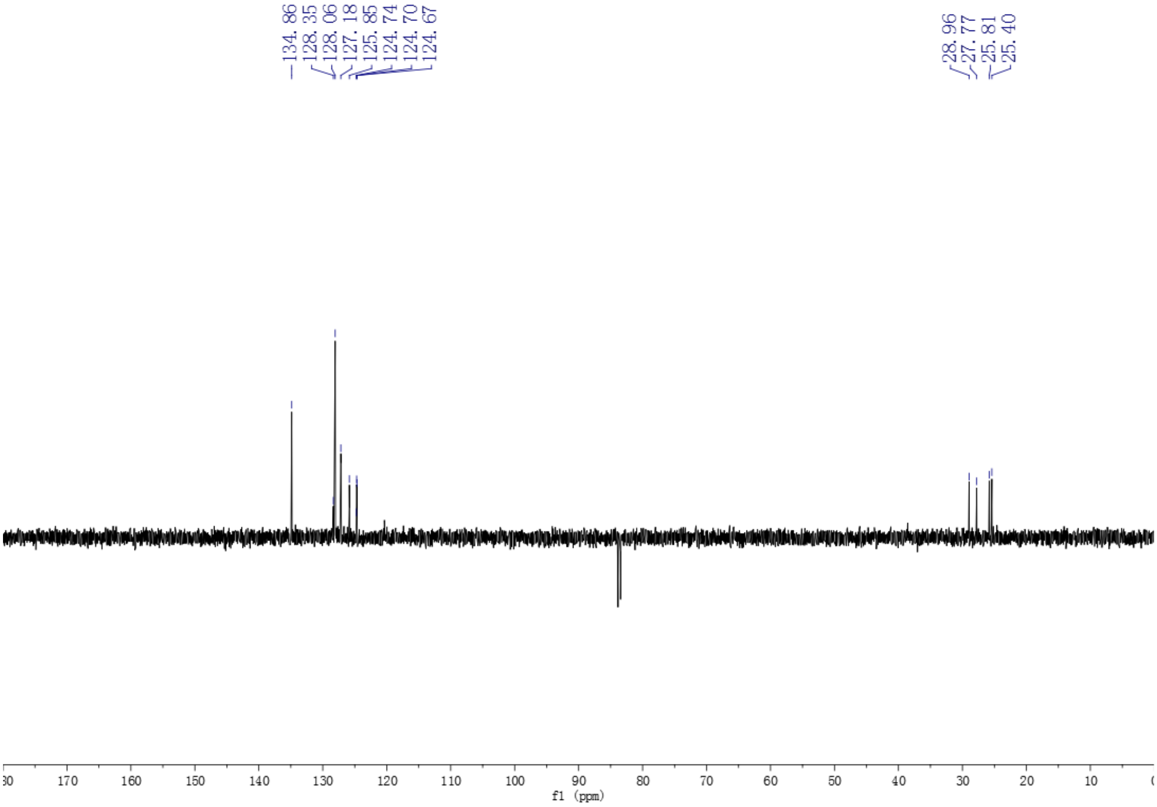

^{11}B NMR

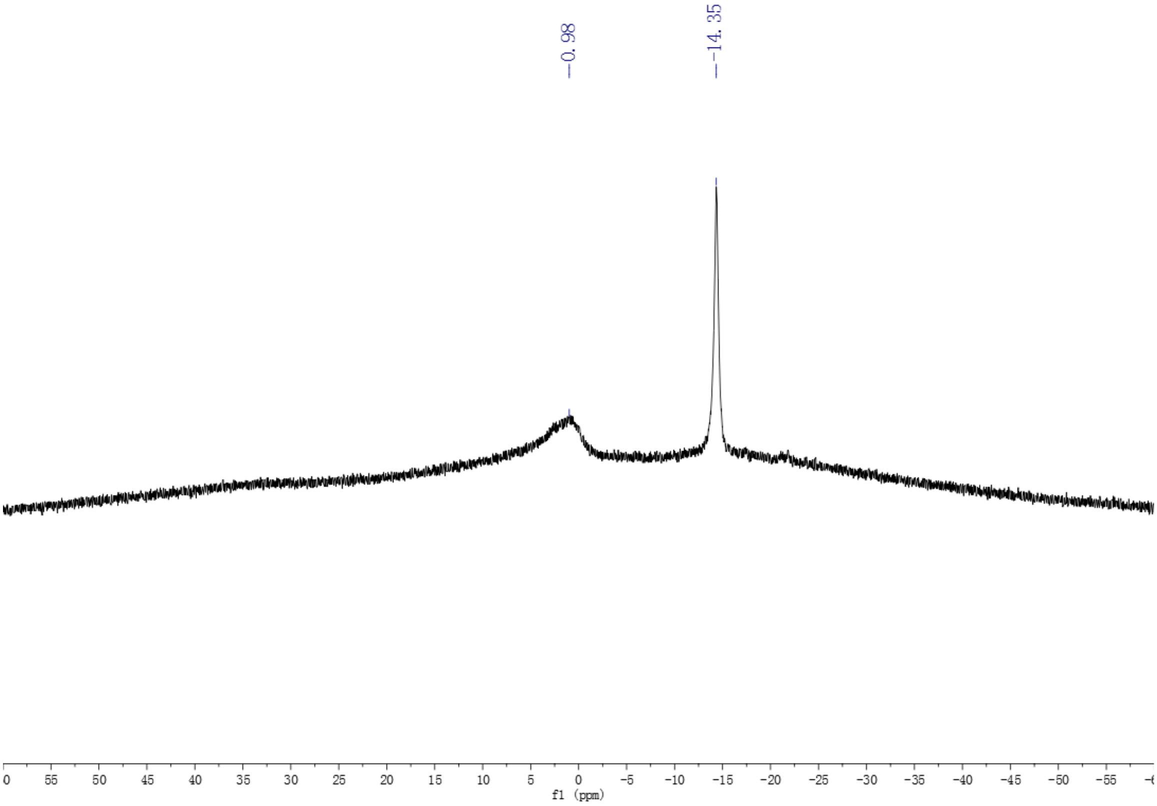

^{19}F NMR

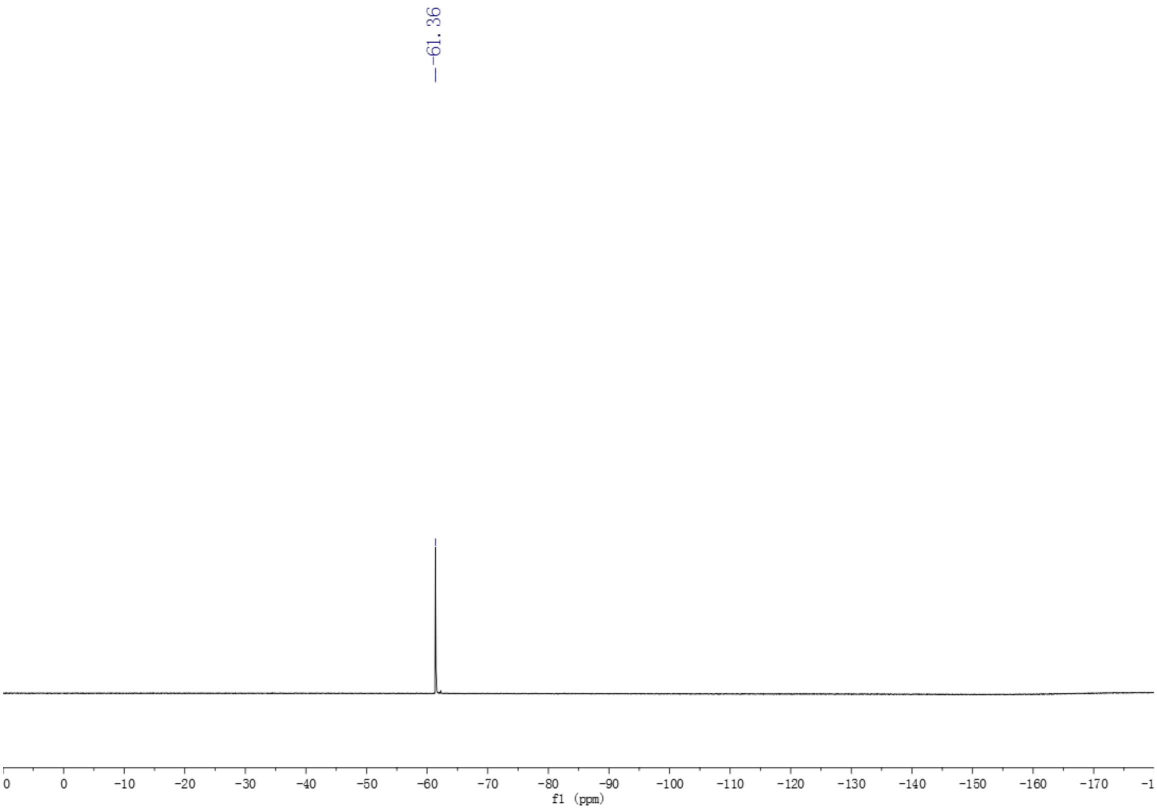

Compound 6

^{1}H NMR

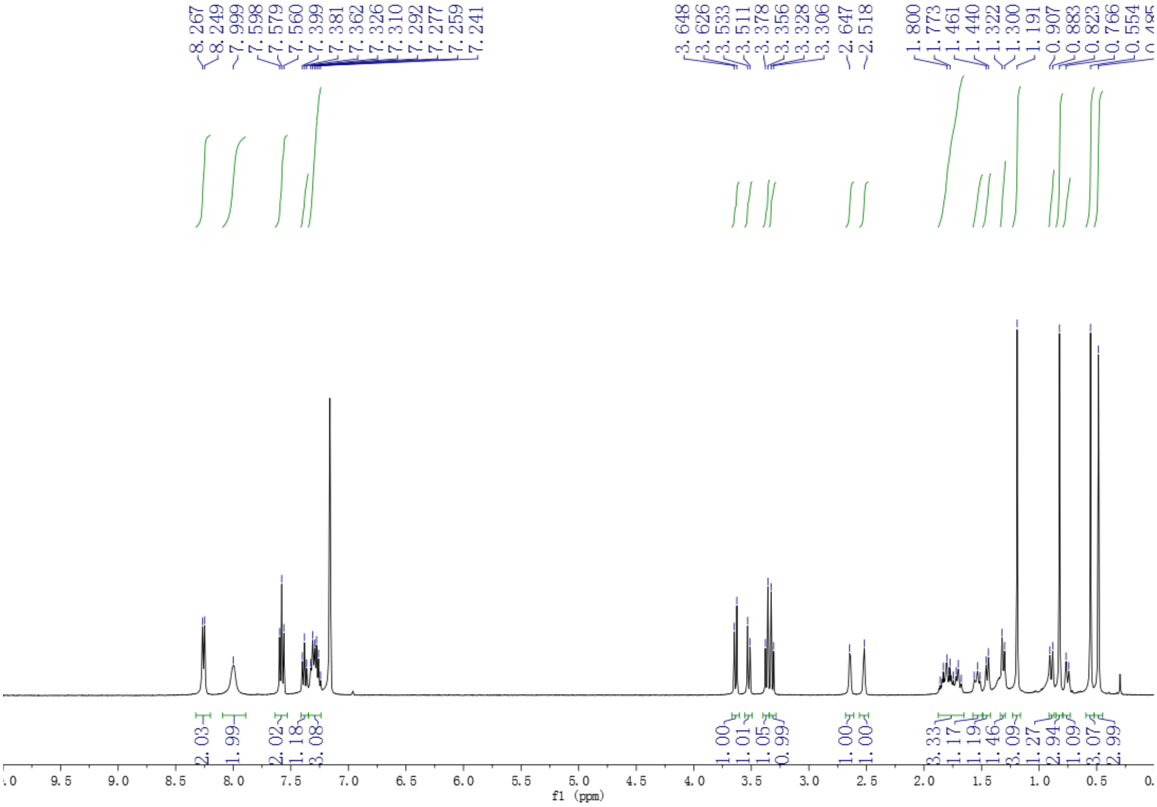

^{13}C NMR

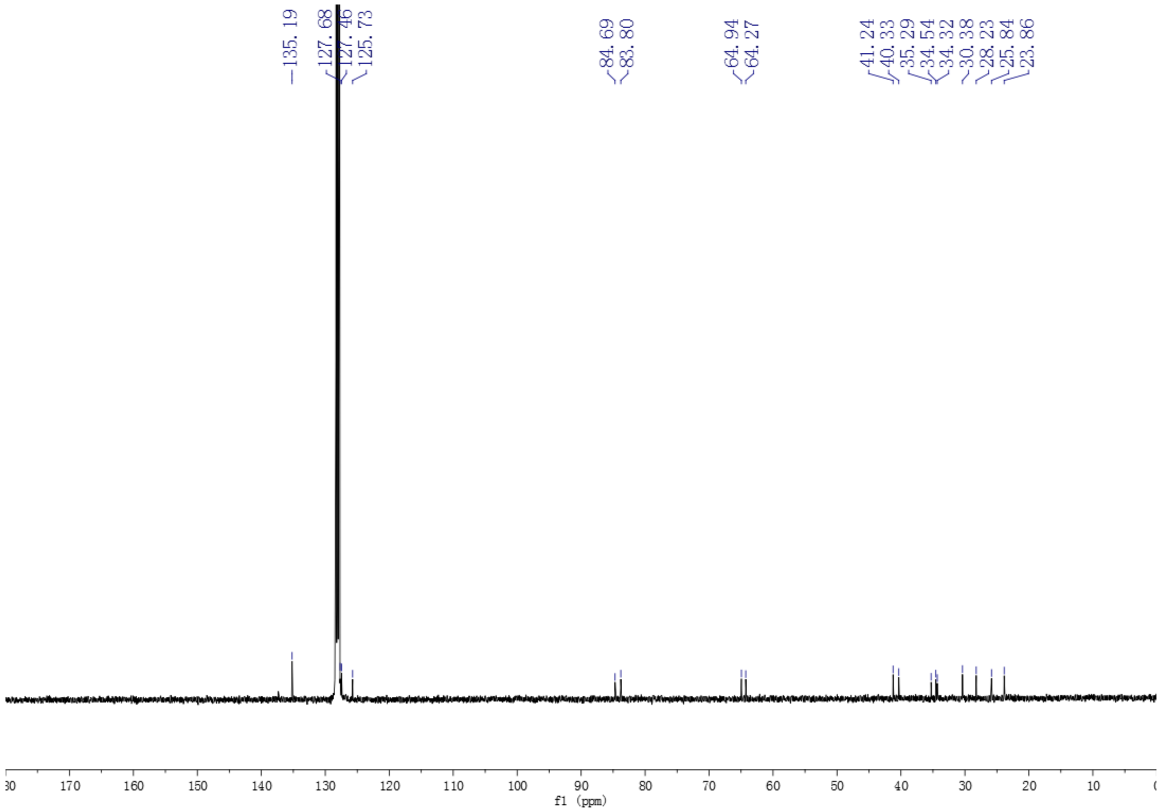

^{13}C NMR (DEPT 135)

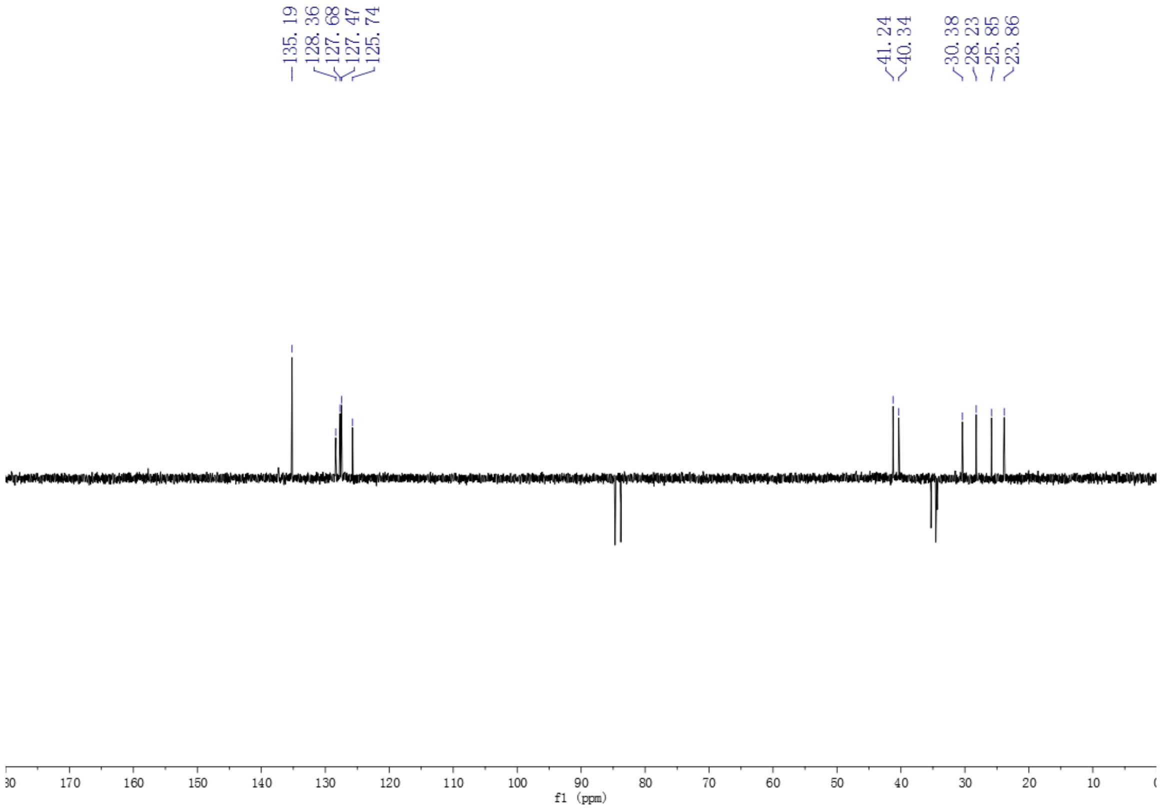

^{11}B NMR

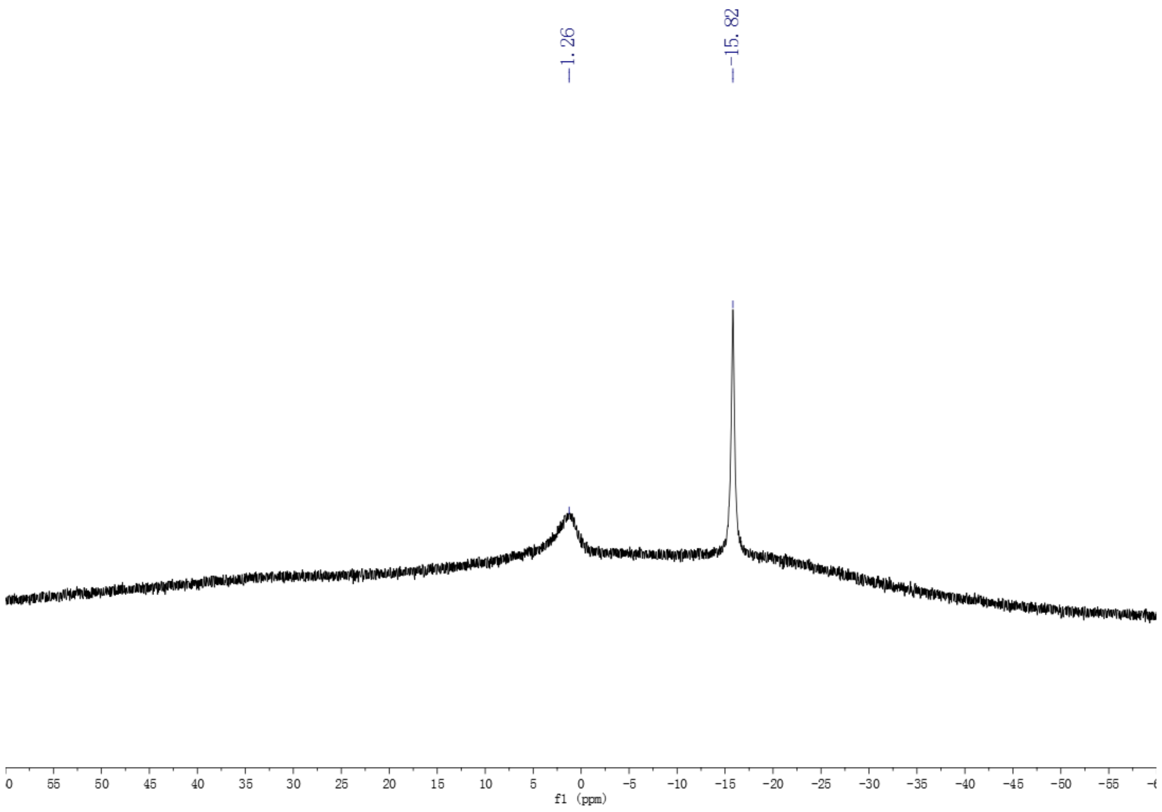

1.26
-15.82
0
55
50
45
40
35
30
25
20
15
10
5
0
-5
-10
-15
-20
-25
-30
-35
-40
-45
-50
-55
f1 (ppm)
